# The E2 ubiquitin-conjugating enzymes UBE2D1 and UBE2D2 regulate VEGFR2 dynamics and endothelial function

**DOI:** 10.1242/jcs.260657

**Published:** 2023-05-25

**Authors:** William R. Critchley, Gina A. Smith, Ian C. Zachary, Michael A. Harrison, Sreenivasan Ponnambalam

**Affiliations:** ^1^ Endothelial Cell Biology Unit, School of Molecular and Cellular Biology, University of Leeds, Leeds LS2 9JT, UK; ^2^ Centre for Cardiovascular Biology & Medicine, Rayne Building, University College London, London WC1E 6JF, UK; ^3^ School of Biomedical Sciences, University of Leeds, Leeds LS2 9JT, UK

**Keywords:** VEGFR2, Endothelial cells, Ubiquitin, UBE2D1, UBE2D2, Signalling, Angiogenesis

## Abstract

Vascular endothelial growth factor receptor 2 (VEGFR2, encoded by *KDR*) regulates endothelial function and angiogenesis. VEGFR2 undergoes ubiquitination that programs this receptor for trafficking and proteolysis, but the ubiquitin-modifying enzymes involved are ill-defined. Herein, we used a reverse genetics screen for the human E2 family of ubiquitin-conjugating enzymes to identify gene products that regulate VEGFR2 ubiquitination and proteolysis. We found that depletion of either UBE2D1 or UBE2D2 in endothelial cells caused a rise in steady-state VEGFR2 levels. This rise in plasma membrane VEGFR2 levels impacted on VEGF-A-stimulated signalling, with increased activation of canonical MAPK, phospholipase Cγ1 and Akt pathways. Analysis of biosynthetic VEGFR2 is consistent with a role for UBE2D enzymes in influencing plasma membrane VEGFR2 levels. Cell-surface-specific biotinylation and recycling studies showed an increase in VEGFR2 recycling to the plasma membrane upon reduction in UBE2D levels. Depletion of either UBE2D1 or UBE2D2 stimulated endothelial tubulogenesis, which is consistent with increased VEGFR2 plasma membrane levels promoting the cellular response to exogenous VEGF-A. Our studies identify a key role for UBE2D1 and UBE2D2 in regulating VEGFR2 function in angiogenesis.

## Teaser

**Summary:** The E2 ubiquitin-conjugating enzymes UBE2D1 and UBE2D2 modulate the levels of VEGFR2, which regulates endothelial function and angiogenesis.

## INTRODUCTION

Angiogenesis is the process through which new blood vessel sprout from pre-existing ones; this is essential for vascular homeostasis, wound healing and revascularisation. Dysregulated angiogenesis is a major factor in disease pathologies, including diabetic retinopathy (Martin et al., 2003), cardiovascular disease (Khurana et al., 2005) and tumour growth (De Palma et al., 2017). Soluble pro-angiogenic factors bind to membrane-bound receptors on the endothelium and activate multiple signalling pathways resulting in angiogenesis. However, we lack details of the mechanism(s) explaining how receptor–ligand dynamics regulate endothelial responses in spite of the identification of many angiogenic regulators. A major pro-angiogenic cytokine, vascular endothelial growth factor A (VEGF-A), that is secreted by many cell types exerts its pro-angiogenic effects by predominantly interacting with VEGFR2 (encoded by *KDR*), a receptor tyrosine kinase (Chung and Ferrara, 2011). VEGF-A stimulates VEGFR2 tyrosine autophosphorylation, receptor internalisation and activation of multiple signal transduction pathways: these events promote endothelial cell migration, proliferation and tubule formation (tubulogenesis) (Ewan et al., 2006; Fearnley et al., 2016).

It is well established that VEGFR2 undergoes proteolysis linked to ubiquitination (Bruns et al., 2010; Duval et al., 2003), but the underlying regulatory mechanism is unclear. Previous studies show a role for the major E1 ubiquitin-activating enzyme UBA1 in regulating VEGFR2 ubiquitination (Smith et al., 2017). Although a variety of E3 ubiquitin ligases, including c-Cbl, βTrCP, RNF121 and Nedd4 (Duval et al., 2003; Maghsoudlou et al., 2016; Murdaca et al., 2004; Sakaue et al., 2017; Shaik et al., 2012), are postulated to target VEGFR2, we have lacked a logical framework that provides a mechanism for VEGFR2 ubiquitination.

Ubiquitination in eukaryotic species usually involves a tripartite system of ubiquitin-modifying enzymes, such as the E1 (ubiquitin-activating), E2 (ubiquitin-conjugating) and E3 (substrate-recognition) enzymes, that work in concert to conjugate ubiquitin onto one or more lysine residues within the protein substrate (Critchley et al., 2018; Scheffner et al., 1995). Membrane proteins clearly undergo ubiquitination, thus programming trafficking and proteolysis, but we have lacked information on E2 ubiquitin-conjugating enzymes that regulate VEGFR2 dynamics. The complexity of the E2 ubiquitin-conjugating enzymes is highlighted by the 38 E2 members that facilitate ubiquitin conjugation to a wide variety of protein substrates.

To address this issue in the context of VEGFR2 ubiquitination and turnover, we developed a microscopy-based reverse genetics screen to evaluate the E2 ubiquitin-conjugating enzyme requirement for VEGFR2 turnover in endothelial cells. Our previous studies established that the E1 enzyme UBA1, but not UBA6, controls VEGFR2 ubiquitination, which impacts on membrane protein trafficking and turnover, leading to further regulation of VEGF-A-stimulated endothelial responses (Smith et al., 2017). Herein, we used a reverse genetics approach to identify E2 ubiquitin-conjugating enzymes that control VEGFR2 levels, downstream signalling and endothelial responses. By screening the human E2 family, we discovered two closely related E2 ubiquitin-conjugating enzymes (UBE2D1 and UBE2D2) required for VEGFR2 ubiquitination. Our findings demonstrate that UBE2D1 and UBE2D2 are required for VEGFR2 ubiquitination and proteolysis, thus modulating downstream VEGF-A-regulated signalling. Such effects impact on VEGF-A-regulated tubulogenesis, a key requirement for angiogenesis.

## RESULTS

### A reverse genetics screen for the E2 family identifies UBE2D1 and UBE2D2 as direct regulators of VEGFR2 levels

To screen the human E2 ubiquitin-conjugating enzyme family for roles in VEGFR2 ubiquitination, we first compiled a comprehensive database of ubiquitin-specific E2 enzymes to include all validated, putative and catalytically inactive forms. To identify the E2 enzymes required for VEGFR2 turnover, we employed a reverse genetics approach using siRNA-based knockdown of each candidate E2 protein. We have previously described a morphological assay to analyse effects on VEGFR2 levels and used a reverse genetics siRNA-based screen to screen the human E2 family (see Materials and Methods) (Smith et al., 2017).

**Fig. 1.**
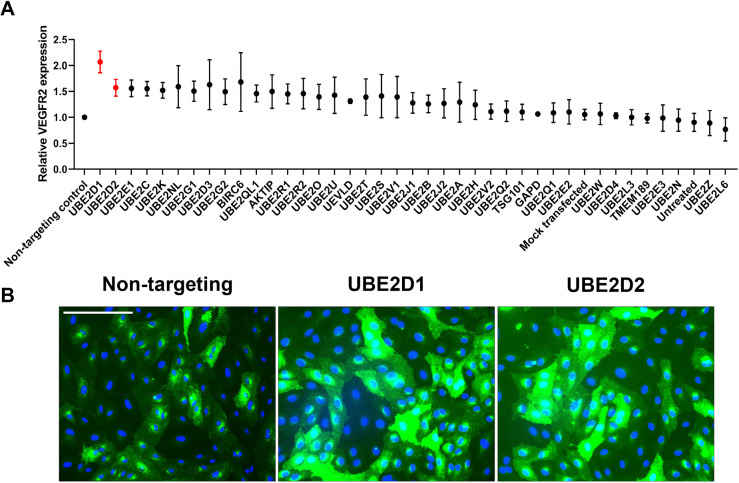
**Reverse genetics screen for the effects of the E2 ubiquitin-conjugating enzyme family on total VEGFR2 levels in endothelial cells.** (A) E2 ubiquitin-conjugating enzyme screen in primary endothelial cells. Histogram quantification of effects of E2 knockdown on total VEGFR2 levels in endothelial cells (see Materials and Methods). For quantification of VEGFR2 expression, a mask was drawn around the entire cytoplasm of each full cell visible in each field of view. The fluorescence intensity of VEGFR2 (green) was measured for a minimum of 50 cells across the three fields of view for each condition using the RGB Measure plugin in ImageJ. The background was subtracted by measuring a representative cell-free region of the field of a similar size. The mean background-subtracted values were then normalised to the mean values for the control siRNA condition to obtain relative VEGFR2 levels. A total of three images per condition were evaluated per experiment, for a total of three independent experiments. Data show the mean±s.e.m. (B) Analysis of total basal VEGFR2 levels in endothelial cells after no transfection, transfection with control non-targeting, UBE2D1-specific or UBE2D2-specific siRNA duplexes for 72 h, followed by immunofluorescence microscopy to detect total VEGFR2 (green) or nuclear DNA (blue). Images are representative of three repeat experiments. Scale bar: 200 µm.

Analysis of VEGFR2 levels in primary human endothelial cells depleted for each of the 38 E2 ubiquitin-conjugating enzymes is depicted in histogram format in Fig. 1A. The most significant effect was a 2-fold rise in VEGFR2 levels caused by UBE2D1 depletion (Fig. 1A,B). Knockdown of a closely related homologue, UBE2D2, caused an ∼1.6-fold rise in VEGFR2 levels (Fig. 1A,B). Interestingly, two other closely related homologues of the UBE2D subfamily, UBE2D3 and UBE2D4, had much smaller effects on VEGFR2 levels (Fig. 1A). Notably, knockdown of UBE2Z, which can only be ubiquitin-primed by UBA6 and not by UBA1, had no effect on VEGFR2 levels, consistent with our previous findings and validating the importance of E1–E2 specificity (Smith et al., 2017).

**Fig. 2.**
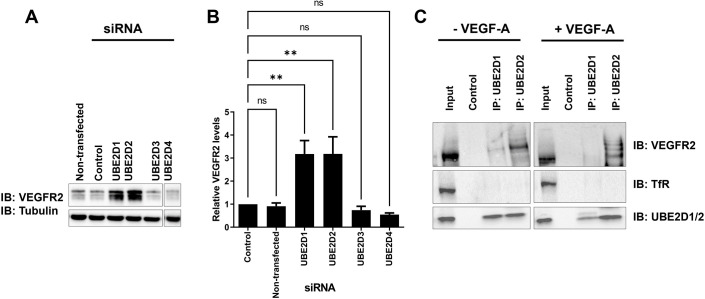
**UBE2D1 and UBE2D2 knockdown stimulates VEGFR2 protein levels in endothelial cells.** (A) Immunoblot analysis of basal total VEGFR2 levels in endothelial cells after treatment with control non-targeting or UBE2D1-, UBE2D2-, UBE2D3- or UBE2D4-specific siRNA duplexes for 72 h. Antibodies against VEGFR2 and tubulin (loading control) were used to analyse protein levels. The triple bands for VEGFR2 represent the immature non-glycosylated form (lower band), partially glycosylated form (middle band) and mature fully glycosylated form (upper band). All bands were quantified together to determine total VEGFR2 levels. (B) Quantification of immunoblotting data of relative VEGFR2 levels in endothelial cells after treatment with control non-targeting or UBE2D1-, UBE2D2-, UBE2D3- or UBE2D4-specific siRNA duplexes (see Materials and Methods). Error bars indicate ±s.e.m. Statistical significance was assessed by one-way ANOVA followed by Tukey's post-test for multiple comparisons. Significance is indicated by asterisks (*n*=3). ns, not significant; **P<0.01. (C) Immunoblot (IB) analysis of co-immunoprecipitation (IP) of VEGFR2 with UBE2D1 and UBE2D2 from HUVEC lysates. Multiple bands were observed for VEGFR2, particularly upon VEGF-A stimulation. Higher molecular mass bands are consistent with and might represent ubiquitinated species of VEGFR2. Transferrin receptor (TfR) was analysed as a control to ensure the specificity of interaction. The polyclonal anti-UBE2D1 antibody was used to detect both UBE2D1 and UBE2D2 (indicated as UBE2D1/2). Owing to the high sequence conservation between UBE2D1 and UBE2D2, polyclonal anti-UBE2D1 also cross-reacts strongly with UBE2D2. Images show the results of a single experiment, performed with and without VEGF-A.

Endothelial cells display variation in steady-state VEGFR2 levels (Fig. 1B). However, knockdown of either UBE2D1 or UBE2D2 as part of the E2 screen caused increased VEGFR2 levels (Fig. 1B). We then compared the effects of knockdown of the UBE2D subfamily, i.e. UBE2D1, UBE2D2, UBE2D3 and UBE2D4, on VEGFR2 levels using immunoblotting (Fig. 2A). UBE2D1 or UBE2D2 knockdown caused a clear rise in VEGFR2 levels, whereas UBE2D3 or UBE2D4 knockdown had no significant effects (Fig. 2A). Quantification of these data showed that knockdown of either UBE2D1 or UBE2D2 produced an ∼3-fold increase in steady-state VEGFR2 levels; however, UBE2D3 or UBE2D4 knockdown had little or no effect compared to the control (Fig. 2B). Such findings support roles for both UBE2D1 and UBE2D2 in regulating VEGFR2 levels in endothelial cells.

As E2 ubiquitin-conjugating enzymes interact with a number of client proteins, we next considered whether VEGFR2 levels were affected directly or indirectly. To address this point, we used antibodies to isolate the UBE2D1 and UBE2D2 complexes from endothelial cell lysates. We detected co-precipitation of VEGFR2 but not transferrin receptor (TfR, encoded by *TFRC*), another plasma membrane receptor, with UBE2D1 and UBE2D2 (Fig. 2C). This occurred in the absence of exogenous VEGF-A (Fig. 2C). VEGFR2–UBE2D2 complex formation was particularly evident (Fig. 2C). Ubiquitinated VEGFR2 species with a higher molecular mass were also evident in both basal and VEGF-A-stimulated conditions followed by UBE2D complex isolation (Fig. 2C).

### UBE2D1 and UBE2D2 regulation of VEGFR2 signal transduction and membrane trafficking

**Fig. 3.**
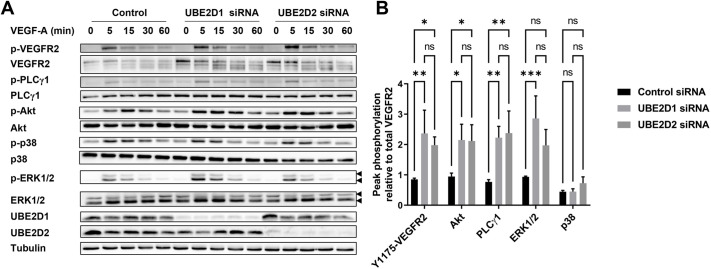
**Elevated VEGFR2 levels caused by UBE2D1 or UBE2D2 knockdown promotes downstream activation and signalling in multiple pathways.** (A) Immunoblot analysis of VEGF-A-stimulated signalling events after transfection with control non-targeting, UBE2D1 or UBE2D2 siRNA. Cells were transfected with siRNAs for 72 h, followed by addition of 25 ng/ml VEGF-A to the culture medium and sample collection at the indicated timepoints. Images are representative of three independent experiments. The triple bands for VEGFR2 represent immature (lower band), partially glycosylated (middle band) and mature fully glycosylated (upper band) forms. The double bands observed for pERK and total ERK represent ERK1 (MAPK3) (upper arrowheads) and ERK2 (MAPK1) (lower arrowheads). Phosphorylated VEGFR2 and phosphorylated PLCγ1 were probed simultaneously on the same membrane. (B) Quantification of immunoblot data for VEGF-A-regulated signal transduction by monitoring maximal levels of phosphorylated VEGFR2, Akt, PLCγ1, p38 and ERK1/2 in primary endothelial cells transfected with control non-targeting, UBE2D1 or UBE2D2 siRNA. Cells were transfected with siRNAs for 72 h, followed by addition of 25 ng/ml VEGF-A to the culture medium, with sample collection at 0, 5, 15, 30 and 60 min. The intensity for each phosphorylated band was quantified at each timepoint using ImageJ pixel density analysis and divided by the intensity of the corresponding band for the total protein, normalised against the intensity of the tubulin control band. All data are shown relative to total VEGFR2. For each replicate, the timepoint at which maximal phosphorylation was observed was recorded and used for analysis (‘peak phosphorylation’). Error bars indicate ±s.e.m. (*n*=3). Significance was determined by two-way ANOVA followed by Dunnett's post-test for multiple comparisons. *P<0.05; **P<0.01; ***P<0.001.

We then asked whether UBE2D1 or UBE2D2-mediated regulation of VEGFR2 levels impacts on VEGF-A-regulated signal transduction pathways (Fig. 3). VEGF-A binding stimulates VEGFR2 tyrosine kinase activity; one characteristic feature is the rapid appearance of the phosphorylated tyrosine epitope (VEGFR2-pY1175) within 5 min of VEGF-A addition, followed by a slower decline in VEGFR2-pY1175 levels (Fig. 3A). Analysis of endothelial cells subjected to UBE2D1 or UBE2D2 knockdown showed an increase in VEGFR2-pY1175 levels (Fig. 3A); this corresponds to a >2-fold increase in VEGFR2-pY1175 levels upon either UBE2D1 or UBE2D2 knockdown compared to those in the control (Fig. 3B). Comparison of different signal transduction pathways showed an ∼2- to 3-fold increase in VEGF-A-stimulated activation of Akt, PLCγ1 and ERK pathways upon either UBE2D1 or UBE2D2 knockdown (Fig. 3B). There was little or no effect on VEGF-A-regulated p38 MAPK activation upon knockdown of either UBE2D1 or UBE2D2 (Fig. 3B). These data support roles for UBE2D1 and UBE2D2 in controlling plasma-membrane VEGFR2 dynamics that impact on VEGF-A-stimulated downstream signalling.

**Fig. 4.**
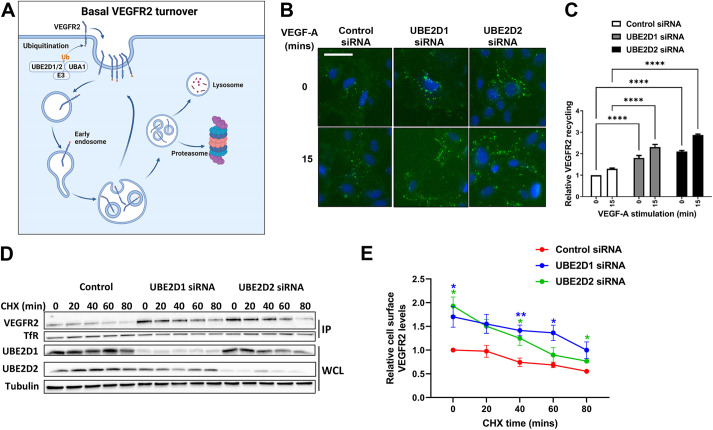
**VEGFR2 recycling between plasma membrane and endosomes depends on UBE2D1 and UBE2D2.** (A) Schematic overview of VEGFR2 internalisation and degradation mediated by basal ubiquitination without stimulation. Created with BioRender.com. (B) Immunofluorescence analysis of VEGFR2 (green) recycling to the plasma membrane after depletion of UBE2D1 or UBE2D2 for 72 h with and without VEGF-A stimulation. Images are representative of three independent experiments. Scale bar: 50 µm. (C) Quantification of relative VEGFR2 recycling from immunofluorescence images in endothelial cells after knockdown with control non-targeting, UBE2D1 or UBE2D2-specific siRNA. The fluorescence intensity of VEGFR2 (green channel) was measured within a mask drawn around each individual cell using ImageJ, for each visible cell in three fields of view, and the background was subtracted by measuring a representative cell-free region in each field. The mean values for each condition were normalised to the mean values for the control siRNA condition at timepoint zero to obtain relative changes in recycling. Significance was determined by two-way ANOVA followed by Dunnett's post-test for multiple comparisons (*n*=3). (D) Analysis of change in plasma membrane VEGFR2 levels determined by cell surface biotinylation assay (IP) after cycloheximide (CHX) treatment and siRNA treatment in endothelial cells. Cells were transfected with control non-targeting, UBE2D1 or UBE2D2 siRNA for 72 h, treated with 20 μg/ml cycloheximide (CHX) for the indicated durations, and subjected to the cell surface biotinylation assay (see Materials and Methods), following which cell lysates were prepared for immunoblotting. IP, immunoprecipitation with NeutraAvidin-agarose beads; WCL, whole-cell lysates. (E) Quantification of relative cell surface VEGFR2 levels in E2-depleted endothelial cells treated with cycloheximide. Significance was determined by two-way ANOVA followed by Dunnett's post-test for multiple comparisons (*n*=4). *P<0.05; **P<0.01; ****P<0.0001.

VEGFR2 undergoes endocytosis and delivery to endosomes, followed by degradation or recycling back to the plasma membrane (Fig. 4A) (Ewan et al., 2006; Gampel et al., 2006; Jopling et al., 2014; Lampugnani et al., 2006). To test whether either UBE2D1 or UBE2D2 knockdown modulates VEGFR2 degradation or recycling, we used an endosome-to-plasma membrane recycling assay that only detects VEGFR2 molecules that have undergone at least one round of endocytosis and recycling back to the plasma membrane (Fig. 4B) (Jopling et al., 2011). Depletion of either UBE2D1 or UBE2D2 caused an ∼1.5- to 2-fold increase in the proportion of VEGFR2 recycled back to the plasma membrane compared to that in control untreated cells or in cells treated with VEGF-A (Fig. 4C). Combining cell-surface-specific VEGFR2 biotinylation with inhibition of new protein synthesis (using cycloheximide) caused an ∼1.5-fold increase in mature VEGFR2 levels at the plasma membrane upon depletion of either UBE2D1 or UBE2D2 (Fig. 4D,E).

**Fig. 5.**
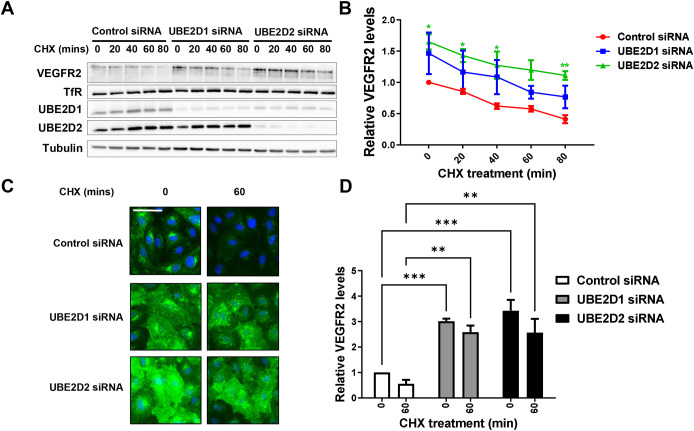
**VEGFR2 levels remain elevated after E2 knockdown despite inhibition of synthesis of new proteins.** (A) Immunoblot analysis of phosphorylated and total VEGFR2 levels after E2 knockdown for 72 h and treatment with cycloheximide for the indicated durations to prevent new protein synthesis, with transferrin receptor (TfR) levels immunoblotted as control. The triple bands observed for VEGFR2 represent immature non-glycosylated (lower band), partially glycosylated (middle band) and fully glycosylated mature (upper band) forms. (B) Quantification of immunoblot data of relative VEGFR2 levels after UBE2D1 and UBE2D2 knockdown and cycloheximide treatment. Significance was determined by two-way ANOVA followed by Dunnett's post-test for multiple comparisons and indicated by asterisks (*n*=3). (C) Analysis of basal VEGFR2 in primary endothelial cells after E2 knockdown for 72 h and cycloheximide (CHX) inhibition of new protein synthesis for the indicated durations as measured by immunofluorescence microscopy to detect VEGFR2 (green) or nuclear DNA (blue). Scale bar: 50 µm. (D) Quantification of immunofluorescence data of relative VEGFR2 levels in primary endothelial cells after control non-targeting, UBE2D1 or UBE2D2 siRNA treatment for 72 h and cycloheximide treatment for the indicated durations (see Materials and Methods). Relative VEGFR2 levels were quantified as described in the legend of Fig. 1A. Error bars indicate ±s.e.m. Significance was determined by two-way ANOVA followed by Dunnett's post-test for multiple comparisons (*n*=3). *P<0.05; **P<0.01; ***P<0.001.

Knockdown of either UBE2D1 or UBE2D2 elevated steady-state or basal VEGFR2 levels; one question was the intracellular location of VEGFR2 ubiquitination. To answer this, we combined cycloheximide treatment (to block biosynthesis of VEGFR2) with UBE2D1 or UBE2D2 knockdown and assessed VEGFR2 levels. In control cells transfected with non-targeting siRNA, we observed a significant decrease in mature VEGFR2 levels (Fig. 5A). In non-stimulated endothelial cells, mature VEGFR2 did not display tyrosine phosphorylation, but underwent degradation over time (Fig. 5A), corresponding to an ∼50% decrease over 80 min (Fig. 5B). Upon knockdown of either UBE2D1 or UBE2D2, mature VEGFR2 levels were initially higher but declined in a similar pattern to those of control (Fig. 5A). For either UBE2D1 or UBE2D2 knockdown, the decrease in mature VEGFR2 levels displayed similar kinetics to those of control (Fig. 5B).

UBE2D1 or UBE2D2 depletion did not affect the levels of the transferrin receptor; this membrane protein recycles between the plasma membrane and endosomes (Fig. 5A). Immunofluorescence microscopy showed that knockdown of UBE2D1 or UBE2D2 followed by cycloheximide treatment to block new protein synthesis caused widespread accumulation of VEGFR2, including at the plasma membrane and in endosomes (Fig. 5C). Quantification of VEGFR2 staining showed an ∼3-fold rise in VEGFR2 levels caused by UBE2D1 or UBE2D2 knockdown, and blocking new protein synthesis using cycloheximide caused only a 10-20% reduction in VEGFR2 levels (Fig. 5D). The knockdown of UBE2D1 and UBE2D2 thus causes mature VEGFR2 accumulation at the plasma membrane and endosomes.

### UBE2D1 and UBE2D2 promote VEGFR2 downregulation to regulate endothelial tubulogenesis

**Fig. 6.**
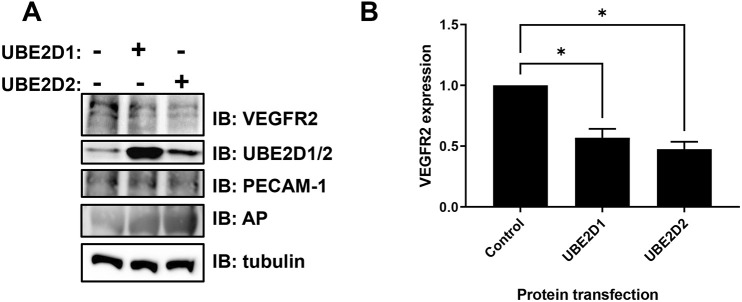
**UBE2D1 and UBE2D2 bind to VEGFR2 and promote downregulation in endothelial cells.** (A) Immunoblot analysis of basal VEGFR2 levels in primary endothelial cells following mock proteofection or proteofection of human recombinant UBE2D1 or UBE2D2 protein for 3 h. Antibodies to VEGFR2, UBE2D1 and UBE2D2 (polyclonal anti-UBE2D1 used to detect both UBE2D1 and UBE2D2, indicated as UBE2D1/2), PECAM-1, alkaline phosphatase (AP) and tubulin (control) were used to analyse protein levels. The three visible bands for VEGFR2 represent immature non-glycosylated (lower band), partially glycosylated (middle band) and mature fully glycosylated (upper band) forms. All forms were quantified together. (B) Quantification of immunoblot data of relative VEGFR2 levels in endothelial cells after mock proteofection (control) or proteofection with UBE2D1 or UBE2D2 recombinant protein. Error bars indicate ±s.e.m. Significance was determined by one-way ANOVA followed by Tukey's post-test for multiple comparisons and indicated by asterisks (*n*=4). *P<0.05.

One likelihood is that the ubiquitin-conjugating enzymes UBE2D1 and UBE2D2 mediate direct conjugation of ubiquitin onto VEGFR2 as a client protein or substrate. To test this idea, we introduced recombinant human UBE2D1 or UBE2D2 proteins directly into endothelial cells using a technique called proteofection (Fig. 6A). Cytoplasmic delivery of recombinant UBE2D1 or UBE2D2 caused an ∼50% decrease in VEGFR2 levels after 3 h (Fig. 6B). These data support roles for both UBE2D1 and UBE2D2 in downregulating VEGFR2 levels in endothelial cells. Cell-surface protein levels of a transmembrane protein (PECAM1) or a glycophosphatidylinositol (GPI)-anchored protein (alkaline phosphatase, AP or ALPL) were not significantly altered by introduction of either UBE2D1 or UBE2D2 into endothelial cells, indicating VEGFR2 specificity.

**Fig. 7.**
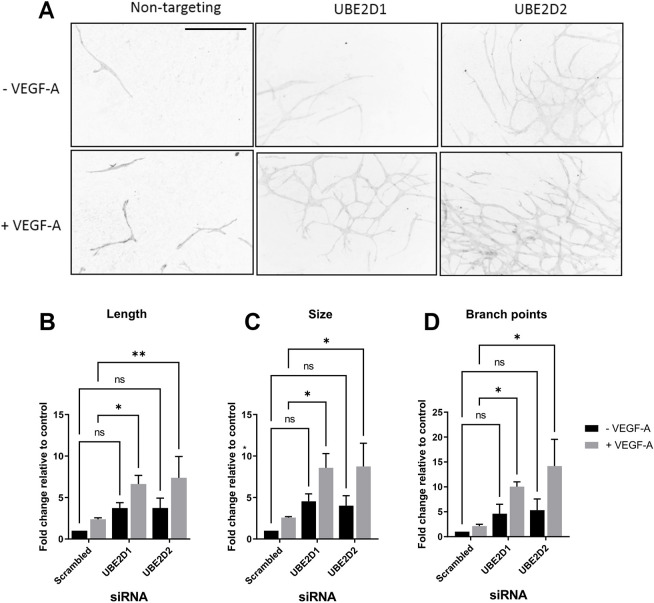
**UBE2D1 and UBE2D2 regulate VEGF-A-stimulated endothelial tubulogenesis.** (A) Analysis of tubule formation using a fibroblast-endothelial co-culture assay after transfection of primary endothelial cells with control non-targeting, UBE2D1 or UBE2D2 siRNA for 72 h in the presence or absence of VEGF-A. Tubules were marked using anti-PECAM-1. Scale bar: 400 μm. (B–D) Quantification of tubulogenesis data for (B) total length, (C) total size and (D) total number of branch points. Three images per condition were analysed for each of three independent experiments. Error bars indicate ±s.e.m. Significance was determined by two-way ANOVA followed by Dunnett's post-test for multiple comparisons (*n*=3). ns, not significant; *P<0.05; **P<0.01.

VEGF-A-stimulated and VEGFR2-regulated signalling normally induces endothelial tubulogenesis, a physiological response that plays a key role in angiogenesis. However, mitogenic signalling is tightly regulated and excessive VEGF-A stimulation can have an inhibitory effect upon angiogenesis (Pontes-Quero et al., 2019). As UBE2D1 or UBE2D2 levels modulate VEGFR2 levels, leading to increased availability and enhanced VEGF-A-regulated signalling, we next asked whether UBE2D1 or UBE2D2 affect VEGF-A-regulated endothelial tubulogenesis. Either UBE2D1 or UBE2D2 depletion caused a substantial increase in endothelial tubulogenesis in both basal and VEGF-A-stimulated conditions (Fig. 7A). Evaluation of endothelial tubule length, tubule size and number of branch points (versus those of controls) revealed that all these parameters increased upon UBE2D1 or UBE2D2 depletion. There was a 4- to 5-fold increase in basal endothelial tubulogenesis (without VEGF-A treatment; Fig. 7B–D). Upon VEGF-A stimulation, there was an ∼3-fold increase in endothelial tubulogenesis following UBE2D1 or UBE2D2 depletion versus that in controls (Fig. 7B–D). These data support roles for UBE2D1 and UBE2D2 in the VEGF-A-regulated endothelial response that contributes to angiogenesis.

## DISCUSSION

VEGFR2 represents a class of membrane proteins with embedded enzymatic activity, i.e. receptor tyrosine kinases, which link ligand binding to signal transduction pathways that control cell function and physiology. Although it is well documented that VEGFR2 undergoes ubiquitination, the nature of the enzymes that regulate VEGFR2 ubiquitination is ill-defined. In this study, we found that two closely related ubiquitin-conjugating E2 enzymes, UBE2D1 and UBE2D2, regulate VEGFR2 ubiquitination and VEGF-A-regulated endothelial function. This is supported by five lines of evidence. First, a reverse genetics screen identified UBE2D1 and UBE2D2 as top-ranked candidates for effects on VEGFR2 levels. Furthermore, depletion of two closely related homologues, UBE2D3 or UBE2D4, had no effects on VEGFR2 levels. Second, depletion of either UBE2D1 or UBE2D2 caused a rise in plasma-membrane VEGFR2 levels that clearly modulated VEGF-A-regulated signalling pathways. There was also a clear increase in VEGF-A-stimulated activation of the MAPK, PLCγ1 and Akt signalling pathways. Third, both UBE2D1 and UBE2D2 formed complexes with VEGFR2 and downregulated VEGFR2 in endothelial cells. Fourth, depletion of either UBE2D1 or UBE2D2, VEGFR2 recycling between the plasma membrane and endosomes was increased. This implies that reduced VEGFR2 ubiquitination due to reduced UBE2D1 or UBE2D2 facilitates increased trafficking from endosomes back to the plasma membrane. Finally, depletion of UBE2D1 or UBE2D2 promoted endothelial tubulogenesis, an essential requirement for angiogenesis. Interestingly, a physiological response (angiogenesis) was sensitised and elevated under both basal and excess VEGF-A conditions in which UBE2D1 or UBE2D2 was depleted.

Previous work shows that VEGF-A stimulates VEGFR2 activation, ubiquitination and degradation (Basagiannis et al., 2017; Bruns et al., 2010; Ewan et al., 2006). However, endothelial cells also utilise a UBA1-dependent pathway to control basal VEGFR2 levels independent of VEGF-A-stimulated VEGFR2 degradation (Smith et al., 2017). Herein, VEGF-A stimulation caused similar levels of VEGFR2 decrease in control, UBE2D1-depleted and UBE2D2-depleted cells, supporting the existence of a separate pathway controlling VEGF-A-stimulated VEGFR2 ubiquitination. One interpretation is that UBA1 working alongside UBE2D1 or UBE2D2 and a hitherto uncharacterised E3 ubiquitin ligase facilitates the transfer of ubiquitin to VEGFR2, which subsequently modulates membrane receptor trafficking and proteolysis. Such regulation influences VEGFR2 bioavailability at the plasma membrane. Our postulated mechanism could have wider implications, as epidermal growth factor receptor (EGFR) is also noted to undergo ubiquitination and degradation (Katz et al., 2002) without tyrosine kinase activation (Opresko et al., 1995).

The enhanced bioavailability of VEGFR2 at the plasma membrane caused by UBE2D1 or UBE2D2 knockdown allows an increase in specific signalling output through PLCγ1, Akt and ERK1/2 phosphorylation. However, it is interesting to note that p38 MAPK activation, a key target of VEGF-A stimulation, was not affected by this rise in VEGFR2 levels. In this context, our previous work demonstrates that p38 MAPK activation is independent of canonical MAPK and PI3K-Akt signalling pathways (Fearnley et al., 2016). Knockdown of clathrin heavy chain CHC17 levels, which blocks clathrin-dependent endocytosis, markedly inhibits VEGF-A-dependent Akt and ERK1/2 activation, but p38 MAPK levels are not affected (Fearnley et al., 2016). These findings highlight clathrin dependence for canonical MAPK and PI3K-Akt signalling, whereas p38 MAPK activation occurs via a different route. In the context of this study, elevated VEGFR2 levels are due to a lack of basal ubiquitination by UBE2D1 and/or UBE2D2: upon VEGF-A stimulation, an increase in clathrin-dependent VEGFR2 endocytosis promotes canonical MAPK and PI3K-Akt signalling events.

The intracellular location of UBE2D-regulated VEGFR2 ubiquitination remains to be determined. One likelihood is that plasma-membrane VEGFR2 levels are increased upon depleted UBE2D1 or UBE2D2 levels associated with the endocytic pathway, i.e. plasma membrane and/or endosomes. Alternatively, biosynthetic VEGFR2 could be targeted for ubiquitination and degradation by an E3 ubiquitin ligase, RNF121 (Maghsoudlou et al., 2016). Thus, although the exact location of UBE2D1- and UBE2D2-mediated VEGFR2 ubiquitination is unclear, this pathway modulates VEGFR2 trafficking in the endosome–lysosome network, which impacts on proteolysis.

One possibility is that UBE2D1 and UBE2D1 interact with UBA1, one of the major E1 ubiquitin-activating enzymes. Previous studies have shown that UBA1 depletion increases plasma membrane VEGFR2 levels, with similar effects on VEGF-A-stimulated signal transduction and tubulogenesis (Smith et al., 2017). UBA1 interactions with UBE2D1 or UBE2D2 are well documented (Jin et al., 2007)= and involved in the ubiquitination of 100–200 client proteins or substrates (https://thebiogrid.org/). Interestingly, we detected a stable UBE2D–VEGFR2 complex in endothelial cells, which likely includes E1 and E3 enzymes. The identity of the E3 ubiquitin ligase in this complex remains unclear. A number of studies suggest a variety of E3 ubiquitin ligases that regulate VEGFR2, including c-Cbl, βTrCP and RNF121 (Duval et al., 2003; Maghsoudlou et al., 2016; Shaik et al., 2012). More work is needed to identify the exact composition and properties of the E1–E2–E3 complex that binds VEGFR2 in endothelial cells.

Both UBE2D1 and UBE2D2 are widely expressed enzymes with a large number of interactions and client substrates linked to different cellular processes. Our work showing roles for UBE2D1 and UBE2D2 in controlling VEGFR2 levels highlights roles for specific E2 ubiquitin-conjugating enzymes in angiogenesis. Although E2 knockdown demonstrates a significant VEGFR2-dependent effect on tubulogenesis, there is also the potential for other signalling pathways to influence this process. It is significant that endothelial tubule formation is also elevated without addition of exogenous VEGF-A in UBE2D1- and UBE2D2-knockdown endothelial cells. Endothelial growth medium contains low levels of VEGF-A (3–5 ng/ml) and other growth factors or hormones of uncertain concentration that are needed for endothelial cell homeostasis and survival. This is likely to result in increased tubulogenesis via signals through both VEGFR2 and other pathways. In E2-depleted cells, these signals are likely to be enhanced at least in part due to elevated VEGFR2 levels. It is interesting to observe, however, that there must be additional targets for these E2s that influence angiogenesis. The identity of these factors and the mechanisms by which they exert this effect remain to be investigated. As a case in point, hypoxia-responsive angiogenesis in skeletal muscle is impaired when UBE2D1 levels are elevated upon TNFα stimulation (Basic et al., 2014). Our findings support a role of UBE2D1 and UBE2D2 as regulators of angiogenesis through a direct effect on VEGFR2 levels and bioavailability at the plasma membrane.

## MATERIALS AND METHODS

### Cell culture and materials

Primary human umbilical vein endothelial cells (HUVECs) were isolated from the umbilical cords collected during elective Caesarean section procedures at Leeds General Infirmary (Leeds, UK) with informed consent (ethical approval reference CA03/020 from Leeds NHS Hospitals Local Ethics Committee). HUVECs were cultured as previously described (Howell et al., 2004). HUVECs, endothelial cell growth medium (ECGM), primary normal dermal human fibroblasts and human recombinant VEGF-A_165_ were obtained from PromoCell (Heidelberg, Germany). MCDB131 medium, OptiMEM, Dulbecco's modified Eagle medium (DMEM) and Alexa Fluor 488-conjugated donkey anti-sheep IgG [1:200 for immunofluorescence (IF), A11-015] were obtained from Thermo Fisher Scientific (Waltham, USA). Antibodies were sourced as follows: goat polyclonal anti-VEGFR2 [1:100 for IF, 1:1000 for immunoblotting (IB), AF357] from R&D Systems, Minneapolis, MN, USA; mouse HRP-conjugated anti-α-tubulin (1:10,000 for IB, HRP-66031) from Proteintech, Rosemont, IL, USA; rabbit antibodies to phosphorylated (Y1175) VEGFR2 (1:1000 for IB, #2478), p38 (1:1000 for IB, #8690), phosphorylated p38 (1:1000 for IB, #9215), Akt (1:1000 for IB, 9272S), phosphorylated Akt (1:1000 for IB, 4060B), PLCγ1 (1:1000 for IB, #5690), phosphorylated PLCγ1 (1:1000 for IB, #2821), ERK1/2 (1:1000 for IB, #4695) and phosphorylated ERK1/2 (1:1000 for IB, #4370) from Cell Signalling Technology, Danvers, MA, USA; mouse anti-PECAM1 (1:500 for IF, 1:3000 for IB, #303102) from BioLegend, San Diego, CA, USA; rabbit monoclonal anti-UBE2D1 [EPR13000(B); 1:5000 for IB, 1:250 for immunoprecipitation (IP), ab176561] and rabbit anti-UBE2D2 [EPR11031(B); 1:5000 for IB, 1:250 for IP, ab155088] from Abcam, Cambridge, UK; rabbit polyclonal anti-UBE2D1 (1:10,000 for IB, PA5-28959) from Thermo Fisher Scientific; and mouse anti-transferrin receptor (1:1000 for IB, sc-65882) from Santa Cruz Biotechnology, Dallas, TX, USA. Purified rabbit anti-alkaline phosphatase antibody (1:500 for IB) was obtained from Andrew Booth (University of Leeds, UK). HRP-conjugated secondary antibodies (anti-goat, 1:5000, 705-035-147; anti-mouse, 1:5000, 715-035-151; anti-rabbit, 1:2500, 711-035-152) were obtained from Stratech Scientific (Newmarket, UK). RIPA buffer with EDTA was obtained from Alfa Aesar (MA, USA). ON-TargetPlus siRNA duplexes were obtained from Horizon Discovery (Cambridge, UK). Pro-DeliverIN protein delivery reagent was obtained from OZ Biosciences (Marseille, France). Protein G agarose beads were obtained from Merck Millipore (Burlington, USA). Cell lysis buffer was obtained from Cell Signalling Technology. Duolink proximity ligation assay kit and cycloheximide were purchased from Sigma-Aldrich (Dorset, UK).

### E2 ubiquitin ligase siRNA library

An E2 ubiquitin-conjugating enzyme database was collated to include all validated, putative and catalytically inactive forms. For the screen, E2 library plates were produced by adding 50 nM SMARTpool siRNA (four duplexes per target) specific for each E2 enzyme to separate wells. A separate 96-well library plate was used for each experiment.

### Screening of E2 knockdown on VEGFR2 levels

HUVECs were reverse transfected in 96-well plates grown in serum-free OptiMEM medium with 0.1 µl per well Lipofectamine RNAiMAX (Thermo Fisher Scientific) and a 50 nM SMARTpool siRNA, specific for an E2 enzyme or non-targeting control. Mock-transfected (treated with Lipofectamine RNAiMAX only) and untreated HUVECs were included as controls. HUVECs were incubated with lipid–siRNA complexes for 6 h at 37°C before replacement of OptiMEM with fresh ECGM. After 72 h, cells were processed for immunofluorescence microscopy.

### Immunofluorescence microscopy

HUVECs were fixed in 3% (w/v) paraformaldehyde followed by brief permeabilisation in 0.1% (w/v) Triton X-100. Cells were incubated overnight at room temperature with primary antibodies in 1 mg/ml bovine serum albumin (BSA) in PBS before addition of DAPI and Alexa Fluor 488- or Alexa Fluor 594-conjugated secondary antibodies for 2 h. Images were acquired using an EVOS FL Auto 2 inverted digital microscope (Thermo Fisher Scientific), with three fields of view obtained per condition. Fluorescence intensity was calculated using ImageJ version 1.46r (National Institutes of Health, USA).

### Co-immunoprecipitation

HUVECs were treated as required prior to washing with ice-cold PBS, flash crosslinking with 0.4% paraformaldehyde for 1 min and lysis with RIPA buffer plus EDTA, supplemented with 1 mM PMSF and protease inhibitor cocktail. Equal amounts of lysate were incubated overnight at 4°C with 1 μg of the appropriate rabbit primary antibodies or without antibodies (control). Protein G agarose beads (Merck Millipore, Darmstadt, Germany) were added and incubated at 4°C for 2 h. Beads were washed three times with 0.1× RIPA buffer plus EDTA, pelleted and resuspended in 20 µl 4× Bolt LDS sample buffer (Thermo Fisher Scientific) for immunoblotting.

### Immunoblotting

HUVECs were treated as required before washing with ice-cold PBS and lysis in 2% (w/v) SDS in PBS supplemented with protease inhibitor cocktail and 1 mM PMSF. The bicinchoninic acid assay was used to quantify protein concentrations, before 25 µg protein per lane was loaded onto 10% or 6–20% gradient SDS-PAGE gels. Gels were run at 110 V for approximately 2 h until sufficiently separated. Proteins were transferred overnight at 4°C onto nitrocellulose membranes at 50 mA current. Membranes were blocked in 5 mg/ml BSA in TBS containing 0.1% Tween-20 (TBS-T), then incubated with primary antibody in 1 mg/ml BSA in TBS-T overnight at 4°C, before incubation with donkey HRP-conjugated secondary antibodies for 1 h at room temperature. Immunoblots were developed by enhanced chemiluminescence and detected on a G:Box imaging system (Syngene, Cambridge, UK). ImageJ was used to quantify pixel intensity and normalised against tubulin as loading control. Uncropped images of immunoblots shown in the figures are provided in Fig. S1.

### Proteofection of primary endothelial cells

Delivery of human recombinant protein was achieved by transfection of 50% confluent HUVECs in 24-well plates. Recombinant protein (1 µg/well) was diluted in PBS to 100 µg/ml before combining with Pro-DeliverIN transfection reagent (2 µl/well) and incubating for 15 min at room temperature. OptiMEM was then added at 100 µl/well before immediately adding dropwise onto growing HUVECs. After 3 h incubation at 37°C, the cells were washed twice with ice-cold PBS before lysis in 2% (w/v) SDS for immunoblotting.

### Endothelial tubulogenesis assay

Fibroblasts were grown to confluency in DMEM supplemented with 10% (v/v) fetal bovine serum, 1 mM sodium pyruvate and 1 mM non-essential amino acids. HUVECs were reverse transfected in a 96-well plate as described above. 24 h after transfection, the HUVECs were trypsinised and 5000 cells per well added onto the fibroblast monolayer in duplicate. The cells were incubated for 7 days at 37°C in a 1:1 ratio of ECGM and DMEM with or without 25 ng/ml VEGF-A_165_ supplementation (added every 2 days). After 7 days growth, the cells were washed with PBS before fixation in 3% (w/v) paraformaldehyde without permeabilisation for immunofluorescence analysis. Anti-PECAM-1 staining was used to allow visualisation of endothelial tubules. Endothelial tubule length, size (representative of tubule area as a function of length and thickness) and number of branch points were quantified using AngioQuant software version 1.33 (Niemisto et al., 2005).

### Plasma membrane VEGFR2 recycling

E2-depleted and control HUVECs were serum starved for 2 h in MCDB131 medium containing 0.2% BSA before incubation in goat anti-VEGFR2 primary antibody for 30 min at 37°C. VEGF-A was then added for an additional 30 min. Cell surface primary antibody was stripped by acid wash with serum-free MCDB131 medium at pH 2 at 4°C, followed by two washes in normal MCDB131 medium. Cells were incubated with donkey anti-sheep Alexa Fluor 488-conjugated secondary antibody for 30 min at 37°C before fixation and DAPI addition. Images were acquired using an EVOS-FL inverted digital microscope. Fluorescence intensity was calculated using ImageJ.

### Cell surface biotinylation

E2-depleted and control HUVECs were serum starved for 2 h in MCDB131 medium containing 0.2% (w/v) BSA prior to stimulation with 20 μg/ml cycloheximide for intervals up to 80 min. HUVECs were then washed twice in ice-cold PBS before cell surface proteins were biotinylated by 45 min incubation at 4°C with 0.25 mg/ml NHS-biotin (Merck Millipore, Darmstadt, Germany) in PBS containing 2 mM CaCl_2_ and 2 mM MgCl_2_. The biotinylation reaction was quenched with a TBS wash before lysis in buffer containing 1% (v/v) NP-40, 50 mM Tris-HCl pH 7.5, 150 mM NaCl and 1 mM PMSF. NeutraAvidin-agarose beads (Thermo Fisher Scientific) were used to isolate biotinylated cell surface proteins overnight at 4°C. The agarose beads were washed three times with NP-40 buffer before proteins were eluted in SDS sample buffer for SDS-PAGE separation and immunoblot analysis.

## Supplementary Material

10.1242/joces.260657_sup1 Supplementary information

## References

[JCS260657C1] Basagiannis, D., Zografou, S., Galanopoulou, K. and Christoforidis, S. (2017). Dynasore impairs VEGFR2 signalling in an endocytosis-independent manner. *Sci. Rep.* 7, 45035. 10.1038/srep45035 28327657 PMC5361198

[JCS260657C2] Basic, V. T., Jacobsen, A., Sirsjo, A. and Abdel-Halim, S. M. (2014). TNF stimulation induces VHL overexpression and impairs angiogenic potential in skeletal muscle myocytes. *Int. J. Mol. Med.* 34, 228-236. 10.3892/ijmm.2014.1776 24820910

[JCS260657C3] Bruns, A. F., Herbert, S. P., Odell, A. F., Jopling, H. M., Hooper, N. M., Zachary, I. C., Walker, J. H. and Ponnambalam, S. (2010). Ligand-stimulated VEGFR2 signaling is regulated by co-ordinated trafficking and proteolysis. *Traffic* 11, 161-174. 10.1111/j.1600-0854.2009.01001.x 19883397

[JCS260657C4] Chung, A. S. and Ferrara, N. (2011). Developmental and pathological angiogenesis. *Annu. Rev. Cell Dev. Biol.* 27, 563-584. 10.1146/annurev-cellbio-092910-154002 21756109

[JCS260657C5] Critchley, W. R., Pellet-Many, C., Ringham-Terry, B., Harrison, M. A., Zachary, I. C. and Ponnambalam, S. (2018). Receptor tyrosine kinase ubiquitination and de-ubiquitination in signal transduction and receptor trafficking. *Cells* 7, 22. 10.3390/cells7030022 29543760 PMC5870354

[JCS260657C6] De Palma, M., Biziato, D. and Petrova, T. V. (2017). Microenvironmental regulation of tumour angiogenesis. *Nat. Rev. Cancer* 17, 457-474. 10.1038/nrc.2017.51 28706266

[JCS260657C7] Duval, M., Bedard-Goulet, S., Delisle, C. and Gratton, J. P. (2003). Vascular endothelial growth factor-dependent down-regulation of Flk-1/KDR involves Cbl-mediated ubiquitination. Consequences on nitric oxide production from endothelial cells. *J. Biol. Chem.* 278, 20091-20097. 10.1074/jbc.M301410200 12649282

[JCS260657C8] Ewan, L. C., Jopling, H. M., Jia, H., Mittar, S., Bagherzadeh, A., Howell, G. J., Walker, J. H., Zachary, I. C. and Ponnambalam, S. (2006). Intrinsic tyrosine kinase activity is required for vascular endothelial growth factor receptor 2 ubiquitination, sorting and degradation in endothelial cells. *Traffic* 7, 1270-1282. 10.1111/j.1600-0854.2006.00462.x 17004325

[JCS260657C9] Fearnley, G. W., Smith, G. A., Abdul-Zani, I., Yuldasheva, N., Mughal, N. A., Homer-Vanniasinkam, S., Kearney, M. T., Zachary, I. C., Tomlinson, D. C., Harrison, M. A. et al. (2016). VEGF-A isoforms program differential VEGFR2 signal transduction, trafficking and proteolysis. *Biol. Open* 5, 571-583. 10.1242/bio.017434 27044325 PMC4874356

[JCS260657C10] Gampel, A., Moss, L., Jones, M. C., Brunton, V., Norman, J. C. and Mellor, H. (2006). VEGF regulates the mobilization of VEGFR2/KDR from an intracellular endothelial storage compartment. *Blood* 108, 2624-2631. 10.1182/blood-2005-12-007484 16638931

[JCS260657C11] Howell, G. J., Herbert, S. P., Smith, J. M., Mittar, S., Ewan, L. C., Mohammed, M., Hunter, A. R., Simpson, N., Turner, A. J., Zachary, I. et al. (2004). Endothelial cell confluence regulates Weibel-Palade body formation. *Mol. Membr. Biol.* 21, 413-421. 10.1080/09687860400011571 15764371

[JCS260657C12] Jin, J., Li, X., Gygi, S. P. and Harper, J. W. (2007). Dual E1 activation systems for ubiquitin differentially regulate E2 enzyme charging. *Nature* 447, 1135-1138. 10.1038/nature05902 17597759

[JCS260657C13] Jopling, H. M., Howell, G. J., Gamper, N. and Ponnambalam, S. (2011). The VEGFR2 receptor tyrosine kinase undergoes constitutive endosome-to-plasma membrane recycling. *Biochem. Biophys. Res. Commun.* 410, 170-176. 10.1016/j.bbrc.2011.04.093 21539813 PMC6103438

[JCS260657C14] Jopling, H. M., Odell, A. F., Pellet-Many, C., Latham, A. M., Frankel, P., Sivaprasadarao, A., Walker, J. H., Zachary, I. C. and Ponnambalam, S. (2014). Endosome-to-plasma membrane recycling of VEGFR2 receptor tyrosine kinase regulates endothelial function and blood vessel formation. *Cells* 3, 363-385. 10.3390/cells3020363 24785348 PMC4092869

[JCS260657C15] Katz, M., Shtiegman, K., Tal-Or, P., Yakir, L., Mosesson, Y., Harari, D., Machluf, Y., Asao, H., Jovin, T., Sugamura, K. et al. (2002). Ligand-independent degradation of epidermal growth factor receptor involves receptor ubiquitylation and Hgs, an adaptor whose ubiquitin-interacting motif targets ubiquitylation by Nedd4. *Traffic* 3, 740-751. 10.1034/j.1600-0854.2002.31006.x 12230472

[JCS260657C16] Khurana, R., Simons, M., Martin, J. F. and Zachary, I. C. (2005). Role of angiogenesis in cardiovascular disease: a critical appraisal. *Circulation* 112, 1813-1824. 10.1161/CIRCULATIONAHA.105.535294 16172288

[JCS260657C17] Lampugnani, M. G., Orsenigo, F., Gagliani, M. C., Tacchetti, C. and Dejana, E. (2006). Vascular endothelial cadherin controls VEGFR-2 internalization and signaling from intracellular compartments. *J. Cell Biol.* 174, 593-604. 10.1083/jcb.200602080 16893970 PMC2064264

[JCS260657C18] Maghsoudlou, A., Meyer, R. D., Rezazadeh, K., Arafa, E., Pudney, J., Hartsough, E. and Rahimi, N. (2016). RNF121 inhibits angiogenic growth factor signaling by restricting cell surface expression of VEGFR-2. *Traffic* 17, 289-300. 10.1111/tra.12353 26602861 PMC4767591

[JCS260657C19] Martin, A., Komada, M. R. and Sane, D. C. (2003). Abnormal angiogenesis in diabetes mellitus. *Med. Res. Rev.* 23, 117-145. 10.1002/med.10024 12500286

[JCS260657C20] Murdaca, J., Treins, C., Monthouel-Kartmann, M. N., Pontier-Bres, R., Kumar, S., Van Obberghen, E. and Giorgetti-Peraldi, S. (2004). Grb10 prevents Nedd4-mediated vascular endothelial growth factor receptor-2 degradation. *J. Biol. Chem.* 279, 26754-26761. 10.1074/jbc.M311802200 15060076

[JCS260657C21] Niemisto, A., Dunmire, V., Yli-Harja, O., Zhang, W. and Shmulevich, I. (2005). Robust quantification of in vitro angiogenesis through image analysis. *IEEE Trans. Med. Imaging* 24, 549-553. 10.1109/TMI.2004.837339 15822812

[JCS260657C22] Opresko, L. K., Chang, C. P., Will, B. H., Burke, P. M., Gill, G. N. and Wiley, H. S. (1995). Endocytosis and lysosomal targeting of epidermal growth factor receptors are mediated by distinct sequences independent of the tyrosine kinase domain. *J. Biol. Chem.* 270, 4325-4333. 10.1074/jbc.270.9.4325 7876194

[JCS260657C23] Pontes-Quero, S., Fernández-Chacón, M., Luo, W., Lunella, F. F., Casquero-Garcia, V., Garcia-Gonzalez, I., Hermoso, A., Rocha, S. F., Bansal, M. and Benedito, R. (2019). High mitogenic stimulation arrests angiogenesis. *Nat. Commun.* 10, 2016. 10.1038/s41467-019-09875-7 31043605 PMC6494832

[JCS260657C24] Sakaue, T., Sakakibara, I., Uesugi, T., Fujisaki, A., Nakashiro, K. I., Hamakawa, H., Kubota, E., Joh, T., Imai, Y., Izutani, H. et al. (2017). The CUL3-SPOP-DAXX axis is a novel regulator of VEGFR2 expression in vascular endothelial cells. *Sci. Rep.* 7, 42845. 10.1038/srep42845 28216678 PMC5317005

[JCS260657C25] Scheffner, M., Nuber, U. and Huibregtse, J. M. (1995). Protein ubiquitination involving an E1-E2-E3 enzyme ubiquitin thioester cascade. *Nature* 373, 81-83. 10.1038/373081a0 7800044

[JCS260657C26] Shaik, S., Nucera, C., Inuzuka, H., Gao, D., Garnaas, M., Frechette, G., Harris, L., Wan, L., Fukushima, H., Husain, A. et al. (2012). SCF(beta-TRCP) suppresses angiogenesis and thyroid cancer cell migration by promoting ubiquitination and destruction of VEGF receptor 2. *J. Exp. Med.* 209, 1289-1307. 10.1084/jem.20112446 22711876 PMC3405505

[JCS260657C27] Smith, G. A., Fearnley, G. W., Abdul-Zani, I., Wheatcroft, S. B., Tomlinson, D. C., Harrison, M. A. and Ponnambalam, S. (2017). Ubiquitination of basal VEGFR2 regulates signal transduction and endothelial function. *Biol. Open* 6, 1404-1415. 10.1242/bio.027896 28798148 PMC5665470

