# Supplementary figures and images for: The E2 ubiquitin-conjugating enzymes UBE2D1 and UBE2D2 regulate VEGFR2 dynamics and endothelial function
Source: J Cell Sci. 2023 May 25;136(10):jcs260657. doi: 10.1242/jcs.260657 (PMC10234107; doi:10.1242/jcs.260657)

Fig. 2A

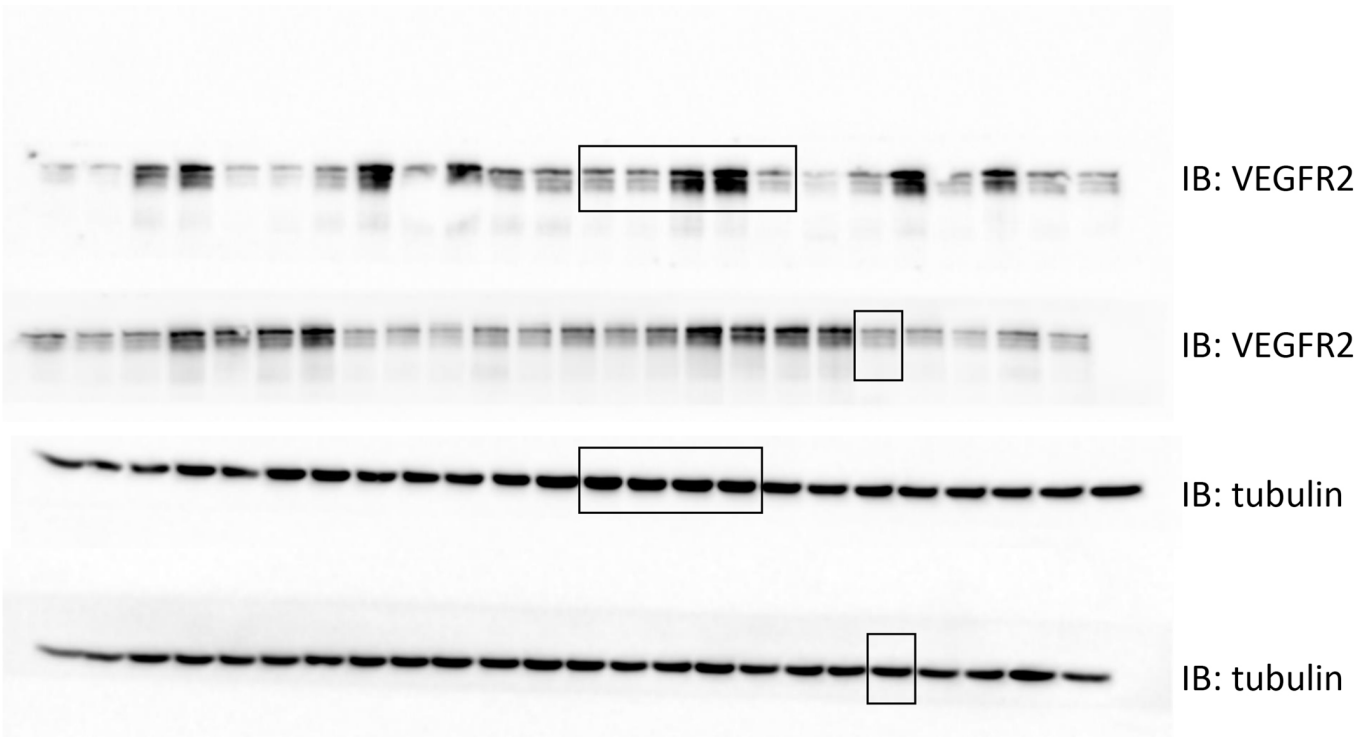

Fig. 2B

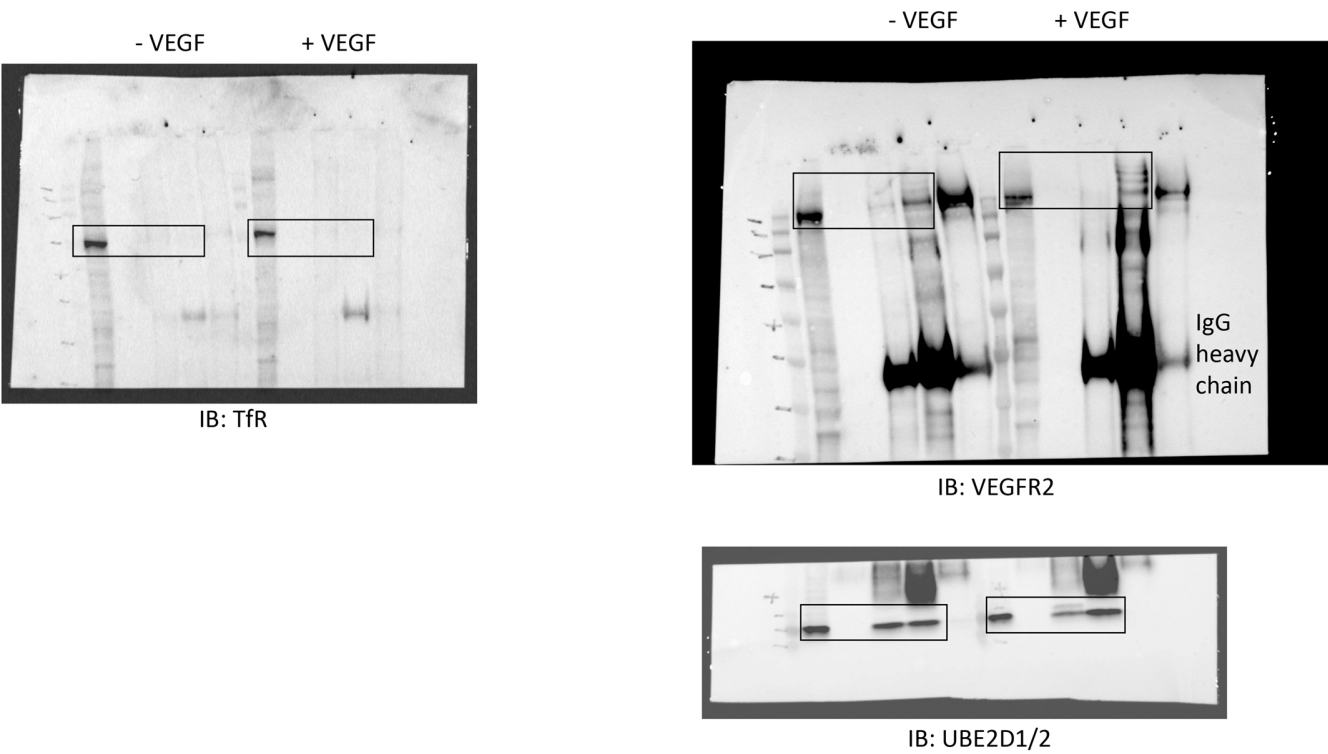

Fig. 3A

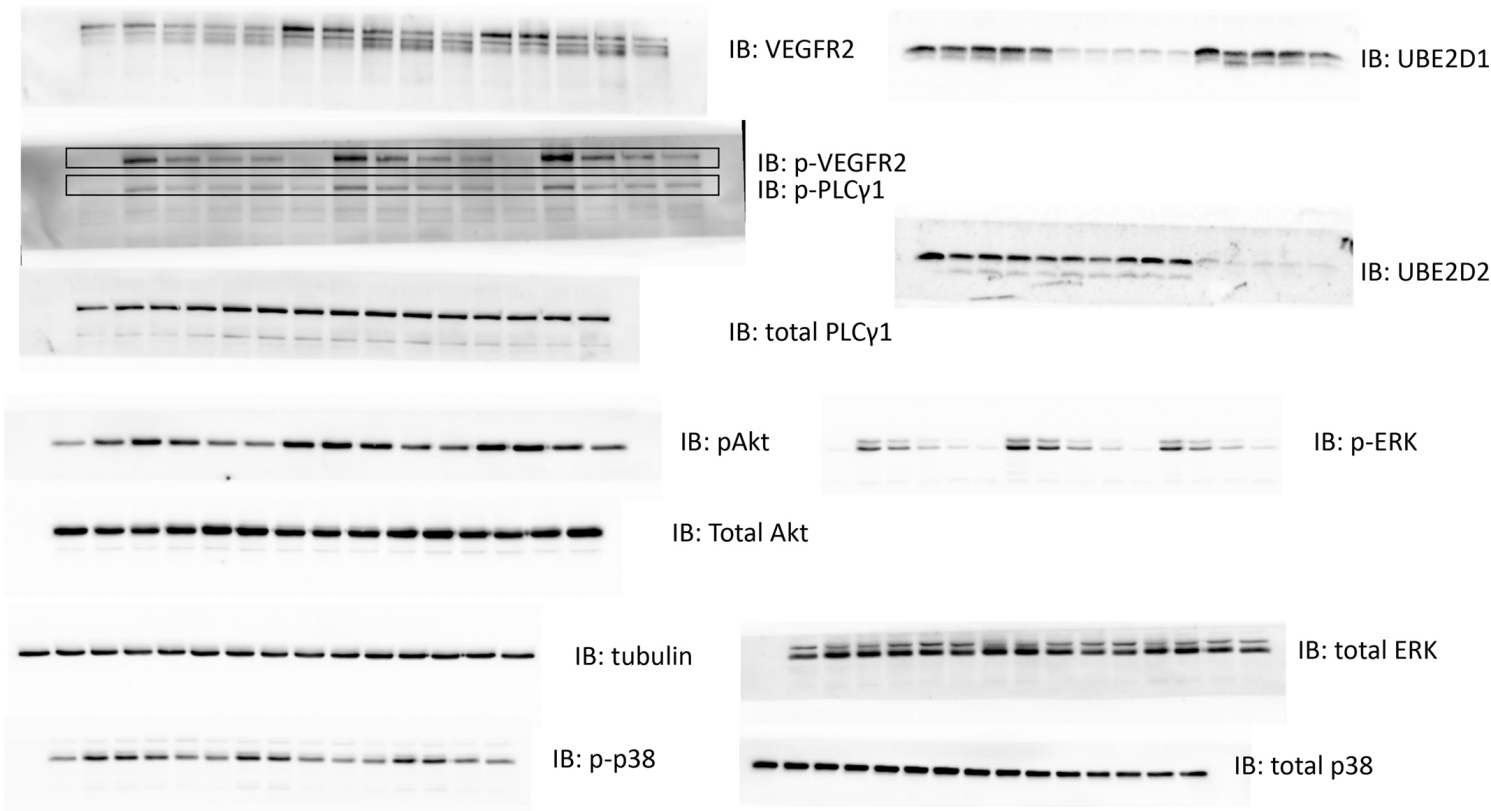

Fig. 4D

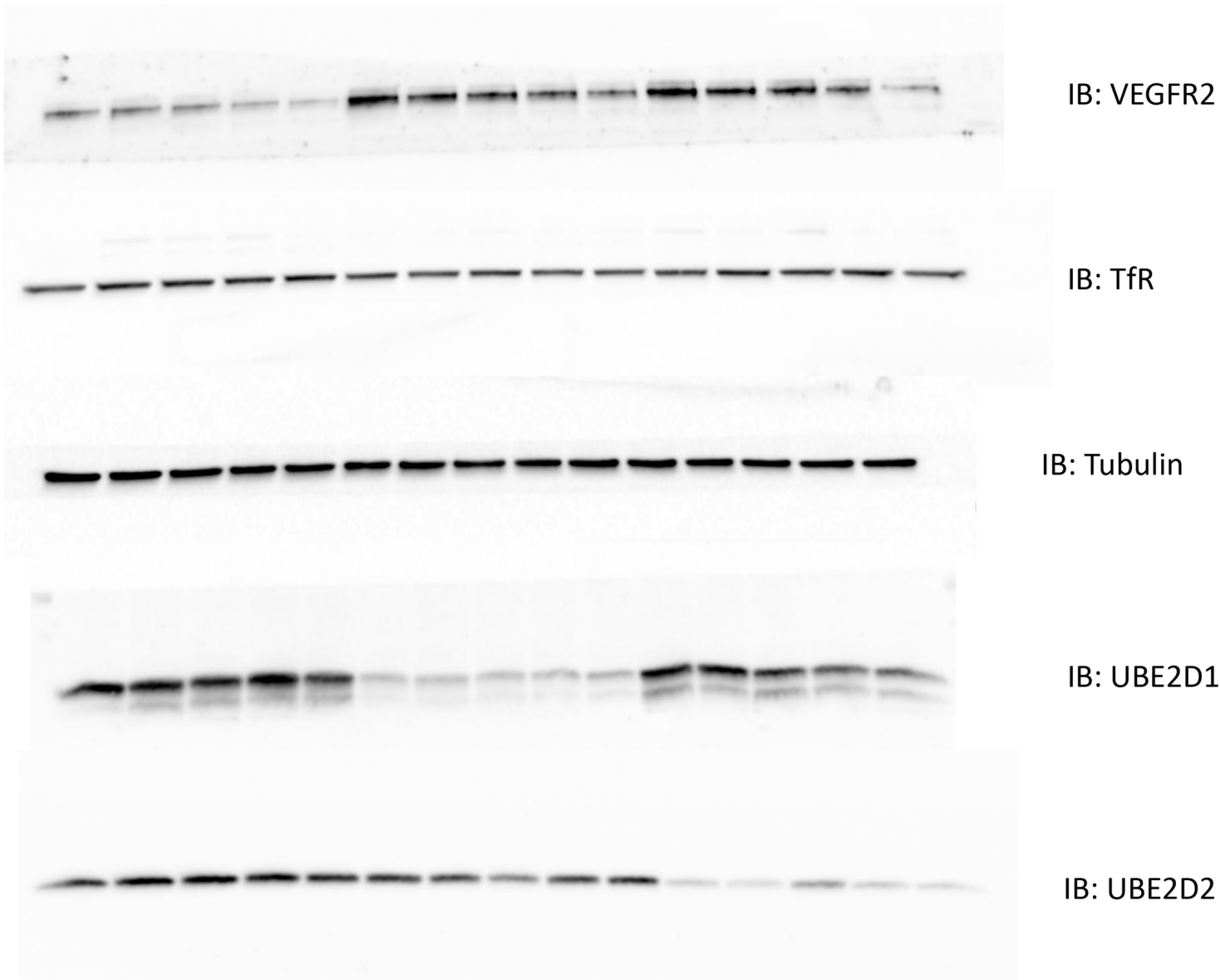

Fig. 5A

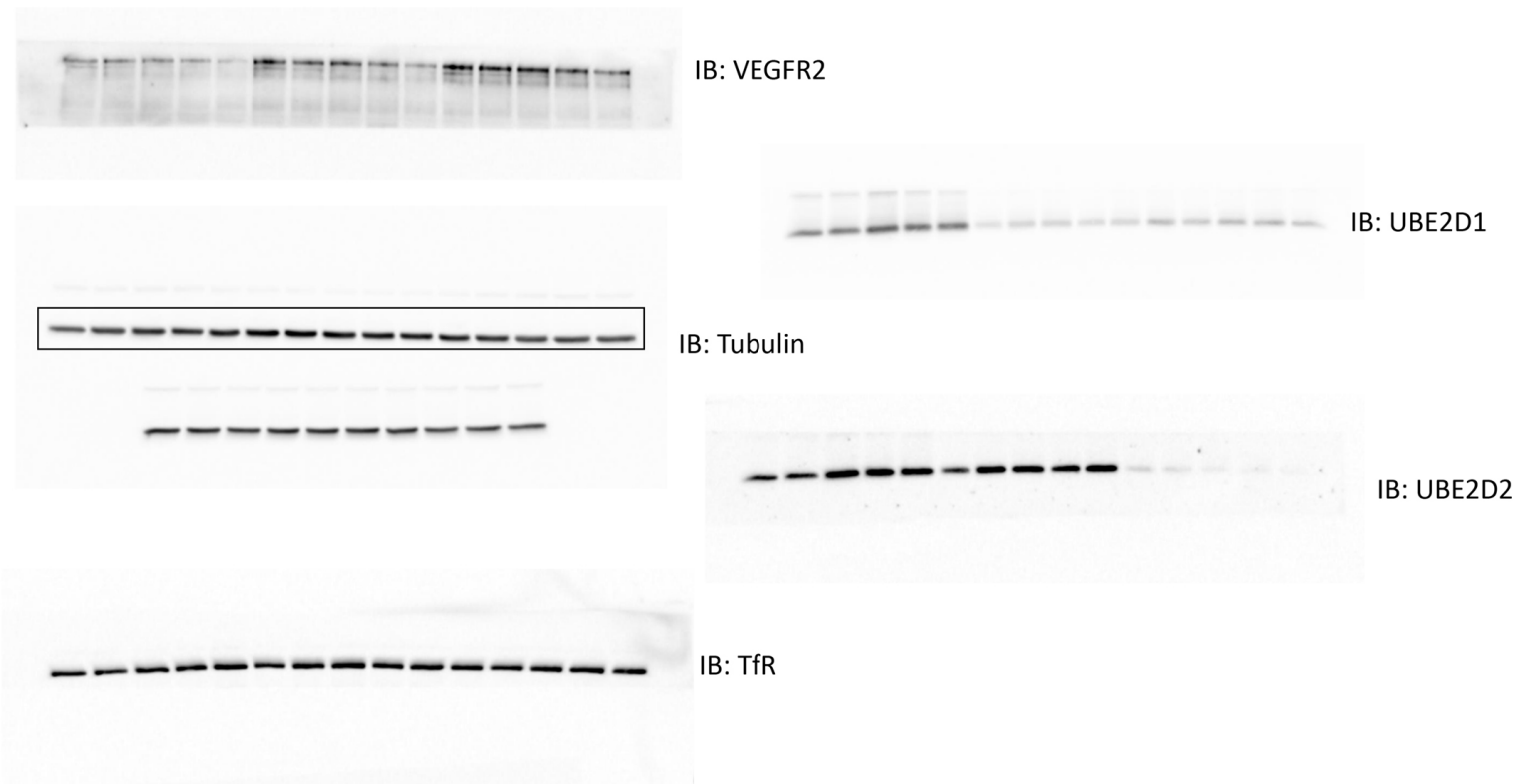

Fig. 6A

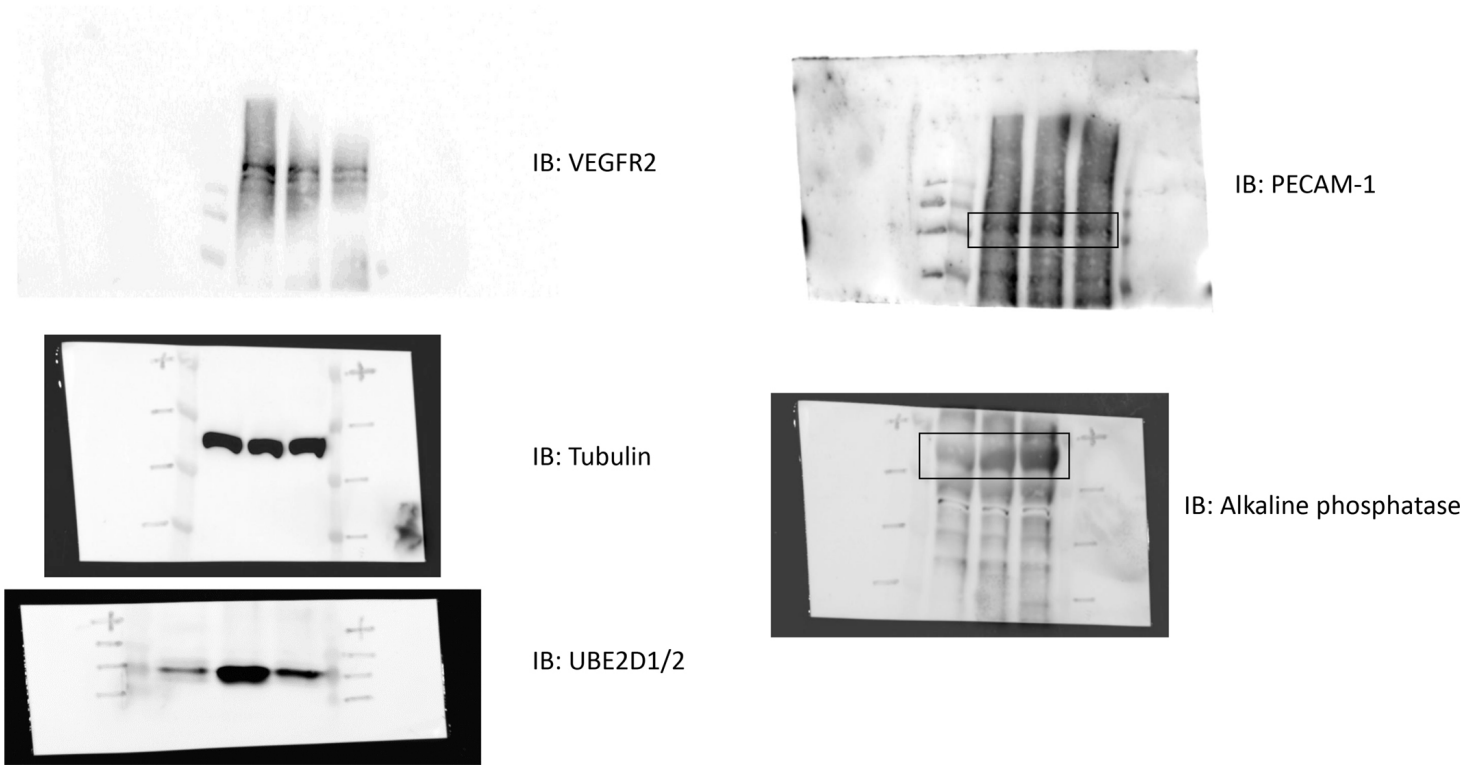

Fig. S1. Blot transparency.

Supplement: Supplementary information [file joces-136-260657-s1.pdf]
