# Supplementary material for: The E2 ubiquitin-conjugating enzymes UBE2D1 and UBE2D2 regulate VEGFR2 dynamics and endothelial function
Source: J Cell Sci. 2023 May 25;136(10):jcs260657. doi: 10.1242/jcs.260657 (PMC10234107; doi:10.1242/jcs.260657)
Supplement: Supplementary file 1 [file joces-136-260657_review_history.pdf]

Journal of Cell Science

The E2 ubiquitin-conjugating enzymes UBE2D1 and UBE2D2 regulate VEGFR2 dynamics and endothelial function

William R. Critchley, Gina A. Smith, Ian C. Zachary, Michael A. Harrison and Sreenivasan Ponnambalam
DOI: 10.1242/jcs.260657

Editor: John Heath

Review timeline

Original submission:	26 September 2022
Editorial decision:	18 November 2022
First revision received:	15 March 2023
Accepted:	5 April 2023

Original submission

First decision letter

MS ID#: JOCES/2022/260657

MS TITLE: E2 ubiquitin-conjugating enzymes, UBE2D1 and UBE2D2, regulate VEGFR2 dynamics and endothelial function

AUTHORS: William Richard Critchley, Gina A Smith, Ian Zachary, Michael A Harrison, and Sreenivasan Ponnambalam
ARTICLE TYPE: Research Article

We have now reached a decision on the above manuscript.

To see the reviewers' reports and a copy of this decision letter, please go to: https://submit-jcs.biologists.org and click on the 'Manuscripts with Decisions' queue in the Author Area. (Corresponding author only has access to reviews.)

As you will see, the reviewers raise a number of substantial criticisms that prevent me from accepting the paper at this stage. They suggest, however, that a revised version might prove acceptable, if you can address their concerns. If you think that you can deal satisfactorily with the criticisms on revision, I would be pleased to see a revised manuscript. We would then return it to the reviewers.

Please ensure that you clearly highlight all changes made in the revised manuscript. Please avoid using 'Tracked changes' in Word files as these are lost in PDF conversion.

I should be grateful if you would also provide a point-by-point response detailing how you have dealt with the points raised by the reviewers in the 'Response to Reviewers' box. Please attend to all of the reviewers' comments. If you do not agree with any of their criticisms or suggestions please explain clearly why this is so.

Reviewer 1

Advance summary and potential significance to field

© 2023. Published by The Company of Biologists under the terms of the Creative Commons Attribution License (https://creativecommons.org/licenses/by/4.0/).

In this study, Critchley et al. has examined expression levels of members of the ubiquitin-conjugating family of proteins in HUVECs and find that UBE2D1 and UBE2D2 are the most highly expressed of the around 40 members. SiRNA-mediated silencing of the corresponding transcripts results in more extensive recycling of VEGFR2 from endosomes to the plasma membrane, where VEGFA-induced signaling becomes more vigorous (although transient) resulting in more enhanced tube formation of the endothelial cells. In agreement overexpression of UBE2D1 and D2 results in decreased cell surface expression of VEGFR2. The presentation is straightforward and seems well controlled and results are adequately evaluated.

Comments for the author

The interest and relevance of this study to the field are unfortunately limited, as there is no in vivo application or disease motivation for the study. More signaling does not necessarily result in the expected increased biological response (more tubules etc). See for example Pontes-Quero et al. Nat Commun 2019 May 1;10(1):2016. doi: 10.1038/s41467-019-09875-7. I understand there may be no animal model available however, siRNA can be delivered also to intact tissues.

Reviewer 2

Advance summary and potential significance to field

The manuscript by Critchley et al investigates the role of Ubiquitin conjugating enzymes in the regulation of VEGFR2 levels. The topic is important and the manuscript imparts novel observations. VEGFR2 is a critical modulator of angiogenesis, a therapeutically active drug target, and detailed knowledge of its regulation is essential. However there are some areas where the experimental approach and or the details surrounding the performed experiment make the data difficult to fully interpret. Similarly, there are several areas where the data does not seem to be internally consistent or lacks important controls for interpretation. Moreover there is an interpretational bias that needs to be addressed. The authors are investigating proteins that modulate ubiquitination and note effects on VEGFR2. There is an implicit bias that this is a direct effect on VEGFR2, rather than indirect effects due to changes in protein levels of other targets that could be essential for maintenance of receptor levels. No data is provided these effects are direct and caution should be urged in presenting the data through this lens. Comments are provided on a Figure by Figure basis and are considered major criticisms unless otherwise noted.

Comments for the author

Figure 1. No methods are available for z score analysis in the Methods sections despite reference in the figure legends. Table S1 is also not available as a supplement, though it is also referenced in the text. Finally the decision to proceed with UBE2D2 is not explained or rationalized. The magnitude of change with this in the screen is no better than many others and the variance appears to be higher (e.g., UBE2C).

Figure 2. The images presented are at a different magnification than other IF images in the manuscript yet methods claimed all were taken at 20X. Legend refers to scale bar that is not present. These data are also total VEGFR2 though that fact is not emphasized or made clear. These data should be accompanied by IF images of non-permeabilized cells, Flow analysis, or surface biotynalation studies to quantify changes in surface levels of VEGFR2. The methods make reference to such experiments being done but this data does not appear to make an appearance anywhere in the manuscript (or the details have been omitted from the figure legends). This figure also seems to be largely redundant with the screening data, seemingly serving as an example. Perhaps the figure could be integrated or replace the phylogenetic tree – the inclusion of which is not well justified.
Figure 3. Comprehensive analysis. Curious that p38 signaling does not seem to be effected. This is a well described response to VEGF stimulus – perhaps this could be discussed (minor).
Figure 4. A persistent problem from this Figure forward is the reporting of "relative" levels in the quantitative data, without reference to how the data is being normalized. The control data in 4C is below 1 so it does not appear to be relative to control. In Figs 4D/E, VEGFR2 is clearly increasing at zero time yet quantitative data does not reflect this, with significant differences noted only with 1 siRNA and only at two time points. Contradicts this data

© 2023. Published by The Company of Biologists under the terms of the Creative Commons Attribution License (https://creativecommons.org/licenses/by/4.0/).

does not align with the statements in the text “Combining cell surface VEGFR2biotinylation with inhibition of new protein synthesis (using cycloheximide) caused ~1.5-foldincrease in mature VEGFR2 levels at the plasma membrane upon depletion of eitherUBE2D1 or UBE2D2 (Figure 4D, 4E)..” This figure legend should reflect that these are biotinylated samples and this experiment needs to be conducted with sufficient repetition to be interpreted quantitatively.
Figure 5. The normalization issue again. It is unclear how these blots are being quantified and normalized. They are not the same blot so it is not (or shouldn’t be) a direct VEGFR2 exp/control calculation. This needs to be clarified. Are there statistics done on the 5B? Figs 5C and D – no subcellular localization of endosomes v plasma membrane is possible given the quality of this figure. This would require confocal with secondary markers. The scale bar is NOT 20 mm as stated in the figure legend. In addition, the quantitative data does not seem to agree with the biochemical data in 5B, as no significant decline is seen in the IF images (even the quantified data), yet in the blots the quantitative data shows a return to near control levels. Given these inconsistencies and limited quality of the images, it is unclear what additional interpretive information this Figure is bringing as currently presented.
Figure 6. This figure is relatively straight forward except for the normalization again. What does relative VEGFR2 levels mean, normalized to vinculin and then controls? Why is vinculin shown rather than tubulin which the authors have been using all along? The UBE2E1/2 proteins are massively overexpressed – have the authors looked at viability under these conditions – its seems possible that given the harshness of this treatment (proteofection) and the nature of the proteins, the downregulation of VEGFR2 could be the result of the cellular toxicity. Blotting with other surface receptors to show specificity could alleviate this criticism.
Figure 7. This Figure presents interesting data but is not fully discussed.
The response to the inhibition of UBE2D1/2, even in the absence of VEGF is robust, especially with UBE2D2, far surpassing the effects of VEGF stimulation.
While there is presumably added VEGF in the ECGM that is part of the basal media, this response seems pharmacologically unusual and is unlikely to be mimicked by enhancing VEGF levels in control cells to occupy more receptors. Do the authors see dose response shifts following siRNA of receptor generated signals? Or other functions such as proliferation? Does an anti-VEGFR2 antibody fully block this response? It seems an untested possibility that there are likely other important targets of this complex that are involved in angiogenesis. The magnitude of the response is also somewhat surprising given that siRNA transfections typically lose their ability to suppress expression after 72 hours or so. These results are 7 days post transfection and its seems unlikely that the VEGFR2 levels are still suppressed. A more detailed analysis could have shed light on some of these uncertainties but at a minimum some of these complexities in interpretation should be addressed in the discussion.

First revision

Author response to reviewers' comments

Reviewer 1
Advance Summary and Potential Significance to Field:

In this study, Critchley et al. has examined expression levels of members of the ubiquitin-conjugating family of proteins in HUVECs and find that UBE2D1 and UBE2D2 are the most highly expressed of the around 40 members. SiRNA-mediated silencing of the corresponding transcripts results in more extensive recycling of VEGFR2 from endosomes to the plasma membrane, where VEGFA-induced signaling becomes more vigorous (although transient) resulting in more enhanced tube formation of the endothelial cells. In agreement, overexpression of UBE2D1 and D2 results in decreased cell surface expression of VEGFR2. The presentation is straightforward and seems well controlled and results are adequately evaluated.

The interest and relevance of this study to the field are unfortunately limited, as there is no in vivo application or disease motivation for the study. More signaling does not necessarily result in the expected increased biological response (more tubules etc). See for example Pontes-Quero et al. Nat

© 2023. Published by The Company of Biologists under the terms of the Creative Commons Attribution License (https://creativecommons.org/licenses/by/4.0/).

Commun 2019 May 1;10(1):2016. doi: 10.1038/s41467-019-09875-7. I understand there may be no animal model available, however, siRNA can be delivered also to intact tissues.

Author reply:

We thank the reviewer for their comments. We acknowledge the lack of disease specific relevance to the study. However, we believe this study to be the first to identify the E2 enzymes involved in regulating VEGFR2 levels at the plasma membrane. As such the study should be of significant interest to colleagues involved in studying angiogenesis (both physiological and pathological) and to the wider scientific community involved in protein turnover and receptor tyrosine kinases. Further evaluation of the mechanistic role of these E2 enzymes in VEGFR2 control in disease settings is certainly necessary but is beyond the scope of this initial study.

We also agree with the reviewer that increased signalling does not always lead to an increased biological response as expected and that excessive signalling may lead to the converse occurrence. We have added a statement to this effect in the manuscript and cited the above reference:

Page 7

'VEGF-A-stimulated and VEGFR2-regulated signalling normally induces endothelial tubule formation (tubulogenesis), a physiological response which plays a key role in angiogenesis. However, mitogenic signalling is tightly regulated and excessive VEGF-A stimulation can have an inhibitory effect upon angiogenesis (Pontes-Quero et al., 2019).'

However, we note that in this particular study, we do indeed demonstrate both enhanced signalling and a corresponding increase in tubular growth in response to loss of UBE2D1 or UBE2D2. We show that E2 knockdown increases surface receptor availability and recycling, allowing greater stimulation by VEGF, leading to enhanced signalling (~2-fold). However, as noted by the reviewer, we observe no alteration in signalling kinetics indicating that stimulation remains broadly at a physiologically pro-angiogenic level not exceeding the upper range of the expected bell-curve response to VEGF.

Regarding in vivo modulation of E2 activity in relation to VEGFR2-regulated angiogenesis, this is a major undertaking that is beyond the scope of this study. We will follow that up in due course, but a number of parameters need to be examined using in vitro and ex vivo cell models before in vivo models are tested.

Reviewer 2 Advance Summary and Potential Significance to Field:

The manuscript by Critchley et al investigates the role of Ubiquitin conjugating enzymes in the regulation of VEGFR2 levels. The topic is important and the manuscript imparts novel observations. VEGFR2 is a critical modulator of angiogenesis, a therapeutically active drug target, and detailed knowledge of its regulation is essential. However there are some areas where the experimental approach and or the details surrounding the performed experiment make the data difficult to fully interpret. Similarly, there are several areas where the data does not seem to be internally consistent or lacks important controls for interpretation. Moreover there is an interpretational bias that needs to be addressed. The authors are investigating proteins that modulate ubiquitination and note effects on VEGFR2. There is an implicit bias that this is a direct effect on VEGFR2, rather than indirect effects due to changes in protein levels of other targets that could be essential for maintenance of receptor levels. No data is provided these effects are direct and caution should be urged in presenting the data through this lens. Comments are provided on a Figure by Figure basis and are considered major criticisms unless otherwise noted.

Author reply:

We thank the reviewer for these very valuable comments that have helped us improve the manuscript. We accept the reviewer's comment that there could be additional client proteins altered by these E2 enzymes that could indirectly influence VEGFR2 maintenance. We certainly agree that these E2 will have multiple interacting partners and that some of these may also be functionally altered by E2 knockdown. We cannot discount the additive effect of E2 knockdown on

© 2023. Published by The Company of Biologists under the terms of the Creative Commons Attribution License (https://creativecommons.org/licenses/by/4.0/).

other proteins involved VEGFR2/angiogenesis. Nonetheless, we have identified a direct interaction between VEGFR2 and these enzymes, with these new data now include (in revised Figure 2C). We also note the co-immunoprecipitation of VEGFR2 with both UBE2D1 and UBE2D2, but not with other negative control protein (transferrin receptor). Thus we have evidence of direct, specific interaction between E2 and VEGFR2. The data are therefore clearly consistent with the idea that direct action of the E2 on VEGFR2 at the very least contributes to the effects we observed. We accept this does not preclude that there may also be other contributing factors.

Reviewer 2:

Figure 1. No methods are available for z score analysis in the Methods sections despite reference in the figure legends.

Author reply:

The method used to calculate the z score was:

Z = (observed value – mean of all values) / standard deviation of all values

However, this was simply an additional representation of the same data shown in Fig. 1B and for the purposes of clarity for the reader we have removed this graph. The simple representation of the mean values (originally Fig 1B) remains.

Reviewer 2:

Table S1 is also not available as a supplement, though it is also referenced in the text.

Author reply:

Apologies – this was thought to be a useful supplement to aid understanding of the study in an earlier draft, but was then removed during rounds of editing/revision before submission – reference to the table was accidentally left in the manuscript, but has not been removed.

Reviewer 2:

Finally the decision to proceed with UBE2D2 is not explained or rationalized. The magnitude of change with this in the screen is no better than many others and the variance appears to be higher (e.g., UBE2C).

Author reply:

We agree with the reviewer that whilst UBE2D1 stood out as an obvious candidate, UBE2D2 had a similar effect to some of the other E2 enzymes in the screen, which may also be worthy of follow up. However, the fact that UBE2D1, UBE2D2 and UBE2D3 were all found to be top 8 hits meant that we decided to focus particularly on the UBE2D family of enzymes for this study because of their relatively close evolutionary relationship. We have in fact also performed an assessment of the effect of UBE2C knockdown on total VEGFR2 levels by immunoblotting and did indeed see an effect on VEGFR2, but to a much lower extent than seen with UBE2D1. We have not included this blot image in the manuscript itself as we have not subsequently followed up this line of investigation.

Reviewer 2:

Figure 2. The images presented are at a different magnification than other IF images in the manuscript yet methods claimed all were taken at 20X. Legend refers to scale bar that is not present. These data are also total VEGFR2, though that fact is not emphasized or made clear. These data should be accompanied by IF images of non-permeabilized cells, Flow analysis, or surface biotynalation studies to quantify changes in surface levels of VEGFR2. The methods make reference to such experiments being done but this data does not appear to make an appearance anywhere in the manuscript (or the details have been omitted from the figure legends). This figure also seems to be largely redundant with the screening data, seemingly serving as an example.

© 2023. Published by The Company of Biologists under the terms of the Creative Commons Attribution License (https://creativecommons.org/licenses/by/4.0/).

Perhaps the figure could be integrated or replace the phylogenetic tree – the inclusion of which is not well justified.

Author reply:

We thank the reviewer for pointing out these inconsistencies.

The phylogenetic tree in the original Figure 1A has been removed as requested.

The missing scale bar has been added back into figure. All scale bars have been checked and corrected as necessary.

The screening data and accompanying follow up blots and IF did not focus specifically on cell surface levels of VEGFR2 as we did not want to miss any important changes in VEGFR2 levels that may occur at other localisations. Once our candidate E2 enzymes had been identified, we then performed cell surface biotinylation studies and recycling assays to discern the impact of E2 knockdown on receptor levels specifically at the plasma membrane (Figure 4). We apologise for not making this clear in the figure legend for Figure 4 and have now edited this accordingly.

As per our reply above, Figure 1 has been altered with the duplicate Z-score graph removed. In this space we have added the IF figures as examples of the images that were obtained from the screen as suggested by the reviewer.

Reviewer 2:

Figure 3. Comprehensive analysis. Curious that p38 signaling does not seem to be effected. This is a well described response to VEGF stimulus – perhaps this could be discussed (minor).

Author reply:

We agree with the referee and have expanded upon this in the discussion on pages 8-9

'The enhanced bioavailability of VEGFR2 at the plasma membrane caused by UBE2D1 or UBE2D2 knockdown allows an increase in specific signalling output through PLCγ1, Akt and ERK1/2 phosphorylation. However, it is interesting to note that p38 MAPK activation, a key target of VEGF-A stimulation is not affected by this rise in VEGFR2 levels. In this context, our previous work demonstrates that p38 MAPK activation is independent of canonical MAPK and PI3K-Akt signalling pathways (Fearnley et al., 2016). Knockdown of clathrin heavy chain CHC17 levels which blocks clathrin-dependent endocytosis markedly inhibits VEGF-A-dependent Akt and ERK1/2 activation, but p38 MAPK levels are not affected (Fearnley et al., 2016). Such findings highlight clathrin-dependence for canonical MAPK and PI3K-Akt signalling, whereas p38 MAPK activation occurs via a different route. In the context of this study, elevated VEGFR2 levels is due to a lack of basal ubiquitination by UBE2D1 and/or UBE2D2: upon VEGF-A stimulation, an increase in clathrin-dependent VEGFR2 endocytosis promotes canonical MAPK and PI3K-Akt signalling events.'

Reviewer 2:

Figure 4. A persistent problem from this Figure forward is the reporting of "relative" levels in the quantitative data, without reference to how the data is being normalized. The control data in 4C is below 1 so it does not appear to be relative to control.
In Figs 4D/E, VEGFR2 is clearly increasing at zero time yet quantitative data does not reflect this, with significant differences noted only with 1 siRNA and only at two time points. Contradicts this data does not align with the statements in the text "Combining cell surface VEGFR2biotinylation with inhibition of new protein synthesis (using cycloheximide) caused ~1.5-foldincrease in mature VEGFR2 levels at the plasma membrane upon depletion of either UBE2D1 or UBE2D2 (Figure 4D, 4E).." This figure legend should reflect that these are biotinylated samples and this experiment needs to be conducted with sufficient repetition to be interpreted quantitatively.

© 2023. Published by The Company of Biologists under the terms of the Creative Commons Attribution License (https://creativecommons.org/licenses/by/4.0/).

Author reply:

The experiment has now been re-analysed to adjust for normalisation to TfR levels at each time point (n=4). Data are now all expressed relative to corresponding 'time zero' control. The graph in the figure has now been updated to reflect the data with corrected normalisation as requested. The blot images have also been replaced with more representative images from the dataset.

Regarding the cell surface biotinylation studies, we have been able to show that UBE2D1 and UBE2D2 knockdown do significantly increase the level of plasma membrane VEGFR2. We accept that in the earlier submission we had not made it sufficiently clear that the data were based on cell surface biotinylation. We have now corrected this.

Reviewer 2:

Figure 5. The normalization issue again. It is unclear how these blots are being quantified and normalized. They are not the same blot so it is not (or shouldn't be) a direct VEGFR2 exp/control calculation. This needs to be clarified. Are there statistics done on the 5B? Figs 5C and D – no subcellular localization of endosomes v plasma membrane is possible given the quality of this figure. This would require confocal with secondary markers. The scale bar is NOT 20 mm as stated in the figure legend. In addition, the quantitative data does not seem to agree with the biochemical data in 5B, as no significant decline is seen in the IF images (even the quantified data), yet in the blots the quantitative data shows a return to near control levels. Given these inconsistencies and limited quality of the images, it is unclear what additional interpretive information this Figure is bringing as currently presented.

Author reply:

In Figure 5B, values for quantitation of VEGFR2 have now been normalised to tubulin to correct for protein gel loading variability. VEGFR2 levels have then been expressed relative to the receptor level in control cells at time point zero. Previously, some of the blots examining the impact of UBE2D1 or UBE2D2 knockdown were done separately, but each had its own non-targeting siRNA control on the same blot to allow for normalisation of the data from that particular blot. Only comparisons for UBE2D1 vs non-targeting and UBE2D2 vs non-targeting were performed. Since the data were usually from different blots, we felt that direct UBE2D1 vs UBE2D2 comparison was not valid. We have changed figure 5 to now show an example in which non-targeting vs UBE2D1 vs UBE2D2 are shown on the same blot, which does make direct comparison possible. Figure 5B has been statistically analysed and the significance is now highlighted in the figure.

Figures 5C and 5D provide a visual overview of the extent of loss of VEGFR2 over time after cycloheximide treatment. The figures are complementary to the blots in Figure 5B showing a more elevated level of VEGFR2 with the loss of E2. The rate of reduction in VEGFR2 level by blotting is similar across each condition, although VEGFR2 levels remain higher throughout when E2 is lost. The scale bar has been corrected in the figure legend.

Reviewer 2:

Figure 6. This figure is relatively straight forward except for the normalization again. What does relative VEGFR2 levels mean, normalized to vinculin and then controls? Why is vinculin shown rather than tubulin which the authors have been using all along? The UBE2E1/2 proteins are massively overexpressed – have the authors looked at viability under these conditions – its seems possible that given the harshness of this treatment (proteofection) and the nature of the proteins, the downregulation of VEGFR2 could be the result of the cellular toxicity. Blotting with other surface receptors to show specificity could alleviate this criticism.

Author reply:

We take on board the reviewer's comment about potential cytotoxicity of the proteofection method. We have now repeated this experiment with analysis of tubulin as loading control, and 2 additional plasma membrane proteins (PECAM1 and alkaline phosphatase) alongside VEGFR2. While we continue to consistently see a decreased level of VEGFR2, neither additional protein was

© 2023. Published by The Company of Biologists under the terms of the Creative Commons Attribution License (https://creativecommons.org/licenses/by/4.0/).

affected by proteofection. We are confident therefore that the method we have used does not cause any more general impact on cell function. These new control data are included in a revised Figure 6, and the text has been amended in the Results section of page 7:
'One likelihood is that the ubiquitin-conjugating enzymes, UBE2D1 and UBE2D2, mediate direct conjugation of ubiquitin onto VEGFR2 as a client protein or substrate. To test this idea, we introduced recombinant human UBE2D1 or UBE2D2 proteins directly into endothelial cells using a technique called proteofection (Figure 6A). Cytoplasmic delivery of recombinant UBE2D1 or UBE2D2 caused ~50% decrease in VEGFR2 levels after 3 h (Figure 6B). These data support roles for both UBE2D1 and UBE2D2 in down-regulating VEGFR2 levels in endothelial cells. Cell surface protein levels of a transmembrane protein (PECAM1) or a GPI-anchored protein (alkaline phosphatase, AP) are not significantly altered by introduction of either UBE2D1 or UBE2D2 into endothelial cells, indicating VEGFR2 specificity.'

Reviewer 2:

Figure 7. This Figure presents interesting data but is not fully discussed. The response to the inhibition of UBE2D1/2, even in the absence of VEGF is robust, especially with UBE2D2, far surpassing the effects of VEGF stimulation. While there is presumably added VEGF in the ECGM that is part of the basal media, this response seems pharmacologically unusual and is unlikely to be mimicked by enhancing VEGF levels in control cells to occupy more receptors. Do the authors see dose response shifts following siRNA of receptor generated signals? Or other functions such as proliferation? Does an anti-VEGFR2 antibody fully block this response? It seems an untested possibility that there are likely other important targets of this complex that are involved in angiogenesis. The magnitude of the response is also somewhat surprising given that siRNA transfections typically lose their ability to suppress expression after 72 hours or so. These results are 7 days post transfection and its seems unlikely that the VEGFR2 levels are still suppressed. A more detailed analysis could have shed light on some of these uncertainties but at a minimum some of these complexities in interpretation should be addressed in the discussion.

Author reply:

The first issue to address is the duration of effect of siRNA knockdown. We are confident that both UBE2D1 and UBE2D2 are at significantly reduced levels for the duration of the tubulogenesis assay. Immunoblots in Figures 3, 4 and 5 for E2 knockdown are with samples at 72 hours post-transfection, and show no evidence of levels starting to increase back towards control levels. In these slow growing primary cells, E2 levels likely remain low for at least a further 48 hours, prolonging the enhancement of VEGFR2 levels for longer.

The second key issue relates to the differences in the relative magnitude of effects on control and E2-depleted cells. We acknowledge that there is a significant effect on these EC that is independent of exogenous VEGF added to the assay system. As the reviewer suggests, this effect is likely caused by a growth factor/hormone background in the culture medium that is essential for EC viability and growth. This effect is unavoidable since the cells simply die without this underlying GF stimulation. In control EC, we typically see an approx. doubling in tubulogenesis outputs caused by additional exogenous VEGF (the data for controls in Fig. 7 are therefore typical of this). The proportional effect of VEGF in E2-depleted cells is broadly in line with the size of this effect in control cells, but of course does not explain the increased tubulogenesis that is apparently VEGF-independent in E2-depleted cells. However, it is worth pointing out that there is increase in VEGF-mediated effects in absolute terms too, consistent with increased signalling input that can only be through VEGFR2. Hence, a proportion of the VEGF-independent effect must be mediated via 'background' signals from growth factor/hormones in the medium (essential for the reason outlined above), but equally a significant proportion of the effects are clearly mediated through increased VEGFR2 levels. It remains an interesting observation that to some extent tubulogenesis (at least in vitro) is specifically influenced by other targets of these E2s, a phenomenon that remains to be investigated further. We have altered the Discussion section to reflect the comments above:

'Both UBE2D1 and UBE2D2 are widely expressed enzymes with a large number of interactions and client substrates linked to different cellular processes. Our work showing roles for UBE2D1 and UBE2D2 in controlling VEGFR2 levels highlights roles for specific E2 ubiquitin-conjugating enzymes in angiogenesis. Whilst E2 knockdown demonstrates a significant VEGFR2 dependent effect on

© 2023. Published by The Company of Biologists under the terms of the Creative Commons Attribution License (https://creativecommons.org/licenses/by/4.0/).

tubulogenesis, there is also the potential for other signalling pathways to influence this process. It is significant that endothelial tubule formation is also elevated without addition of exogenous VEGF-A in UBE2D1 and UBE2D2 knockdown endothelial cells. Endothelial growth medium contains low levels of VEGF-A (3-5ng/ml) and other growth factors/hormones of uncertain concentration that are needed for endothelial cell homeostasis and survival. This is likely to result in increased tubulogenesis via signals through both VEGFR2 and other pathways. In E2 depleted cells, these signals are likely to be enhanced at least in part due to elevated VEGFR2 levels. It is interesting to observe however that there must be additional targets for these E2s that influence angiogenesis. The identity of these factors and the mechanisms by which they exert this effect remain to be investigated.'

Second decision letter

MS ID#: JOCES/2022/260657

MS TITLE: E2 ubiquitin-conjugating enzymes, UBE2D1 and UBE2D2, regulate VEGFR2 dynamics and endothelial function

AUTHORS: William Richard Critchley, Gina A Smith, Ian Zachary, Michael A Harrison, and Sreenivasan Ponnambalam
ARTICLE TYPE: Research Article

I am happy to tell you that your manuscript has been accepted for publication in Journal of Cell Science, pending standard ethics checks.

Reviewer 1

Advance summary and potential significance to field

This reviewer still finds this study of limited interest due to the lack of complex models.

Comments for the author

The authors respond to my comment that they acknowledge the lack of disease relevance. This is correct, but there is also no developmental relevance. The in vivo relevance is not demonstrated. It could very well be that in vivo, in the intact vasculature, UBE2D1 and UBE2D2 play entirely different roles from what the authors observe (upregulation of VEGFR2 expression in HUVECs silenced for UBE2D1 or UBE2D2) and potentially in an organ and vessel type specific manner. I suggest to take out "endothelial function" from the title and change it to "E2 ubiquitin-conjugating enzymes, UBE2D1 and UBE2D2 negatively regulate VEGFR2 expression". This would more accurately reflect the authors' findings.

Reviewer 2

Advance summary and potential significance to field

This reports a novel and potentially significant regulatory mechanism for a major angiogenic factor that is critical for developmental and pathological responses and which is the target of approved therapeutics.

Comments for the author

I appreciate the thorough and careful response of the team to the original critique. The manuscript is markedly improved and I have no further comments.

© 2023. Published by The Company of Biologists under the terms of the Creative Commons Attribution License (https://creativecommons.org/licenses/by/4.0/).
